# Mechanism of RORα in promoting osteoarthritis through p53 deubiquitination-mediated chondrocyte ferroptosis

**DOI:** 10.1016/j.isci.2026.116939

**Published:** 2026-07-27

**Authors:** Lang Mai, Ruijue Zhu, Yankui Liu, Jingxuan Feng, Mudan Huang, Mingyu Yin, Jiawei Di, Lei He

**Affiliations:** 1 Department of Spine Surgery, The Third Affiliated Hospital, Sun Yat-sen University, No. 600 Tianhe Road, Tianhe District, Guangzhou, Guangdong 510630, China; 2 Department of Spine Surgery, The Third Affiliated Hospital of Sun Yat-sen University, Yuedong Hospital, No. 124 Gongyuanbei Road, Meixian District, Meizhou, Guangdong 514700, China; 3 Guangdong Provincial Center for Quality Control of Minimally Invasive Spine Surgery, No. 600 Tianhe Road, Guangzhou 510630, China; 4 Guangdong Provincial Center for Engineering and Technology Research of Minimally Invasive Spine Surgery, No. 600 Tianhe Road, Guangzhou 510630, China; 5 Department of Rehabilitation Medicine, The Third Affiliated Hospital, Sun Yat-sen University, No. 600 Tianhe Road, Tianhe District, Guangzhou, Guangdong 510630, China

**Keywords:** osteoarthritis, chondrocyte ferroptosis, retinoic acid-related orphan receptor alpha, p53

## Abstract

Osteoarthritis (OA) is a degenerative joint disease characterized by progressive cartilage loss, in which iron dysregulation and ferroptosis contribute to disease progression. Here, we investigated the role of the RORα-p53 axis in regulating chondrocyte ferroptosis during OA. Transcriptomic analysis and experimental models revealed activation of ferroptosis and iron imbalance in OA cartilage, while pharmacological inhibition of ferroptosis alleviated cartilage damage. We identified p53 as a key mediator promoting ferroptotic signaling in chondrocytes and demonstrated that retinoic-acid-related orphan receptor alpha (RORα) acts upstream by stabilizing p53. Mechanistically, RORα recruits the deubiquitinase HAUSP to inhibit p53 ubiquitination, thereby enhancing ferroptotic responses. These findings define a regulatory pathway linking RORα to p53-dependent ferroptosis and cartilage degeneration and provide a potential molecular target for disease-modifying OA therapy.

## Highlights

• Ferroptosis is activated in OA cartilage and promotes degeneration
• p53 drives chondrocyte ferroptosis and OA progression
• RORα aggravates OA via p53-dependent ferroptotic signaling
• RORα recruits HAUSP to stabilize p53 through deubiquitination

## Graphical abstract

**Figure:**
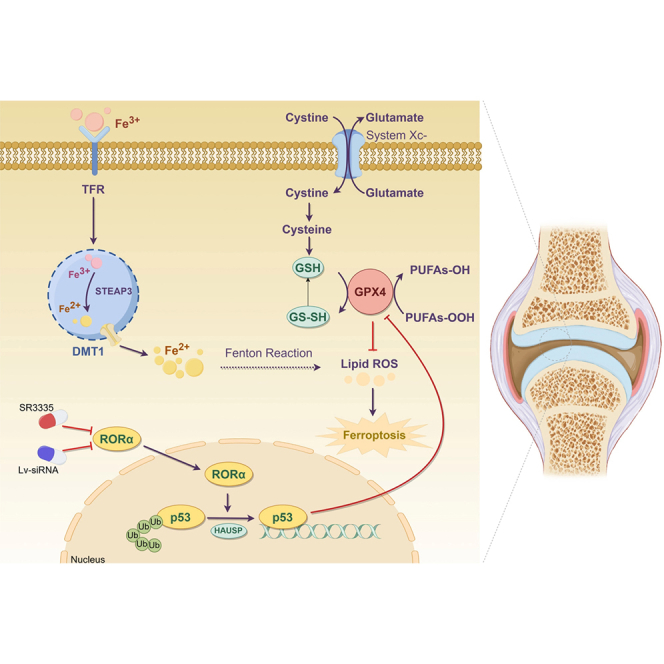

## Teaser

Biological sciences

## Introduction

Osteoarthritis (OA) is the most prevalent chronic joint disease and a leading cause of pain, disability, and reduced quality of life in aging populations worldwide.^1^^,^^2^ Rather than a simple “wear-and-tear” disorder, OA is now recognized as a whole-joint disease involving articular cartilage degeneration, synovial inflammation, subchondral bone remodeling, altered mechanotransduction, and metabolic dysregulation.^3^^,^^4^^,^^5^ Despite substantial advances in understanding disease heterogeneity, current treatment strategies remain largely symptom-oriented and fail to halt structural progression, underscoring the urgent need to identify disease-modifying molecular targets.^6^^,^^7^^,^^8^

Increasing evidence indicates that metabolic imbalance is a central feature of OA pathogenesis. In particular, disturbances in iron homeostasis have emerged as an important pathogenic component linking oxidative stress to cartilage degeneration.^9^^,^^10^^,^^11^ Clinical and experimental studies have shown abnormal iron accumulation in osteoarthritic joints, while elevated systemic iron indices, including serum ferritin, are associated with OA incidence and severity.^12^^,^^13^^,^^14^ Excessive iron promotes reactive oxygen species generation through Fenton chemistry, enhances lipid peroxidation, and sensitizes chondrocytes to stress-induced death, suggesting that iron dyshomeostasis is not merely a secondary consequence of cartilage injury but an active driver of disease progression.^15^^,^^16^^,^^17^

Ferroptosis is an iron-dependent form of regulated cell death characterized by overwhelming phospholipid peroxidation, glutathione depletion, impaired GPX4 activity, and ultrastructural mitochondrial shrinkage.^18^^,^^19^^,^^20^ In recent years, ferroptosis has been increasingly implicated in OA, particularly in articular chondrocytes, which are highly sensitive to redox imbalance because of their limited antioxidant buffering capacity and unique metabolic environment. Hallmark features of ferroptosis, including reduced SLC7A11/GPX4 signaling, elevated lipid ROS, mitochondrial cristae loss, and iron overload, have been detected in osteoarthritic cartilage and experimental OA models.^21^^,^^22^ Notably, Zhang et al. provided early direct evidence that chondrocyte ferroptosis contributes to OA progression and that pharmacological inhibition of ferroptosis alleviates cartilage damage *in vivo*.^23^ More recent reviews and systems-level analyses have further positioned ferroptosis as a central node linking inflammation, oxidative stress, lipid metabolism, and cartilage destruction in OA.^24^^,^^25^^,^^26^ Nevertheless, the upstream regulatory machinery that determines ferroptotic susceptibility in OA chondrocytes remains incompletely understood.

Among candidate upstream regulators, the tumor suppressor protein p53 is of particular interest. Beyond its canonical roles in cell-cycle arrest, senescence, and apoptosis, p53 has emerged as an important modulator of ferroptosis, in part through repression of SLC7A11 and disruption of cystine/glutathione metabolism.^27^^,^^28^^,^^29^ Although p53 signaling has been implicated in cartilage degeneration and chondrocyte stress responses, its role in OA remains incompletely resolved and may depend on disease stage, cellular context, and the balance among apoptosis, senescence, and ferroptosis.^30^ Clarifying how p53 is stabilized and activated in osteoarthritic chondrocytes is therefore essential for understanding its contribution to OA pathology.

Retinoic-acid-related orphan receptor alpha (RORα) is a nuclear receptor involved in circadian regulation, lipid metabolism, inflammation, and skeletal homeostasis.^31^^,^^32^ Previous work has shown that RORα contributes to OA pathogenesis through the CH25H-CYP7B1-RORα axis, linking abnormal cholesterol metabolism to matrix catabolism and cartilage degeneration.^33^^,^^34^^,^^35^ In another biological context, RORα has been shown to stabilize p53 in a HAUSP/USP7-dependent manner, revealing a potential noncanonical role for RORα in post-translational stress signaling.^36^^,^^37^^,^^38^ However, whether RORα regulates p53 stability in osteoarthritic chondrocytes, and whether this mechanism influences ferroptotic vulnerability, remains unknown.

In the present study, we integrated human cartilage specimens, primary chondrocytes, transcriptomic profiling, and chondrocyte-specific RORα conditional knockout mice to investigate the role of ferroptosis in OA and its upstream regulatory mechanism. We demonstrate that ferroptosis is markedly activated in OA, that p53 functions as a key executor of chondrocyte ferroptosis, and that RORα promotes OA progression by stabilizing p53 through HAUSP-dependent deubiquitination. These findings identify the RORα-HAUSP-p53 axis as a previously unrecognized pathogenic pathway linking metabolic dysregulation to ferroptosis-driven cartilage degeneration and suggest a potential therapeutic target for disease-modifying OA intervention.

## Results

### Ferroptosis dysregulation drives OA pathogenesis

**Figure 1:**
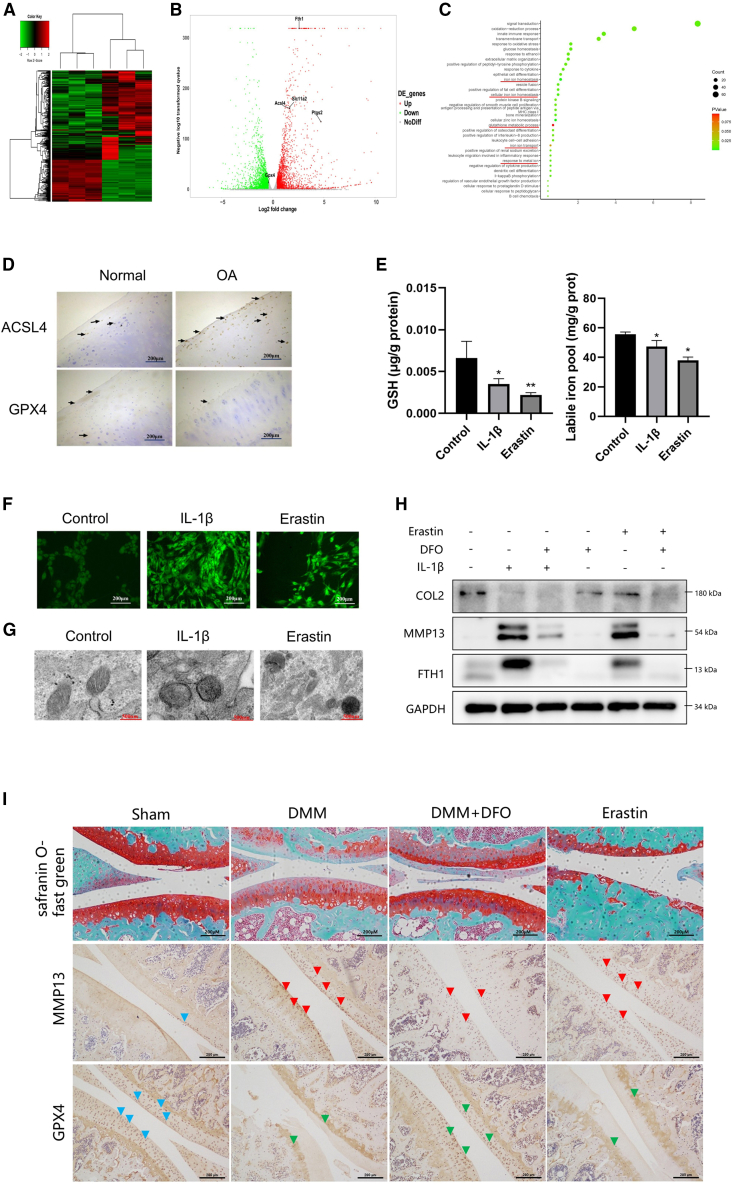
Ferroptosis is activated in osteoarthritic chondrocytes and contributes to cartilage degeneration (A) Heatmap of whole-transcriptome sequencing comparing normal chondrocytes and OA chondrocytes. (B) Volcano plot showing differentially expressed ferroptosis-related genes. (C) GO enrichment analysis indicating dysregulation of iron homeostasis and glutathione metabolism in OA. (D) Immunohistochemical (IHC) staining of human OA cartilage and mouse cartilage tissues showing ACSL4 and GPX4 expression. Scale bars, 200 μm. (E) GSH levels and Fe^2+^ accumulation in IL-1β-treated chondrocytes. (F) Lipid peroxidation levels in IL-1β-induced OA chondrocytes. Scale bars, 200 μm. (G) Transmission electron microscopy images showing mitochondrial shrinkage, increased membrane density, and reduced cristae. Scale bars, 500 nm. (H) Western blot analysis of ferroptosis- and OA-related proteins in chondrocytes treated with DFO. (I) Safranin-O/fast green staining and IHC analysis of mouse knee cartilage following DMM surgery with or without DFO or erastin treatment. Scale bars, 200 μm. Data are representative images or expressed as mean ± SD of each group from at least three experiments; unpaired two-tailed Student’s *t* test (E). ∗*p* < 0.05, ∗∗*p* < 0.01.

Transcriptomic profiling of normal chondrocytes and OA chondrocytes revealed a distinct ferroptosis-associated gene signature (Figure 1A). Key ferroptosis suppressors, including GPX4, was markedly downregulated, whereas ferroptosis-promoting genes such as ACSL4 and FTH1 were significantly upregulated (Figure 1B). Gene Ontology (GO) analysis further identified disrupted iron homeostasis and glutathione metabolism as central pathways in OA progression (Figure 1C).

These alterations were validated at the protein level, as immunohistochemistry of human OA cartilage showed increased ACSL4 and decreased GPX4 expression (Figure 1D). Functionally, interleukin (IL)-1β or erastin treatment induced hallmark ferroptotic features, including glutathione depletion (Figure 1E), increased lipid peroxidation (Figure 1F), and mitochondrial damage characterized by membrane condensation and cristae loss (Figure 1G).

Pharmacological inhibition of ferroptosis with deferoxamine (DFO) reversed these changes *in vitro*, reducing MMP13 and FTH1 while restoring GPX4 and COL2A1 expression (Figure 1H). Consistently, DFO attenuated cartilage degeneration in the DMM mouse model, whereas erastin accelerated cartilage destruction (Figure 1I).

Collectively, these results demonstrate that ferroptosis is actively induced and drives OA pathogenesis.

### P53 mediates ferroptotic signaling in chondrocytes

**Figure 2:**
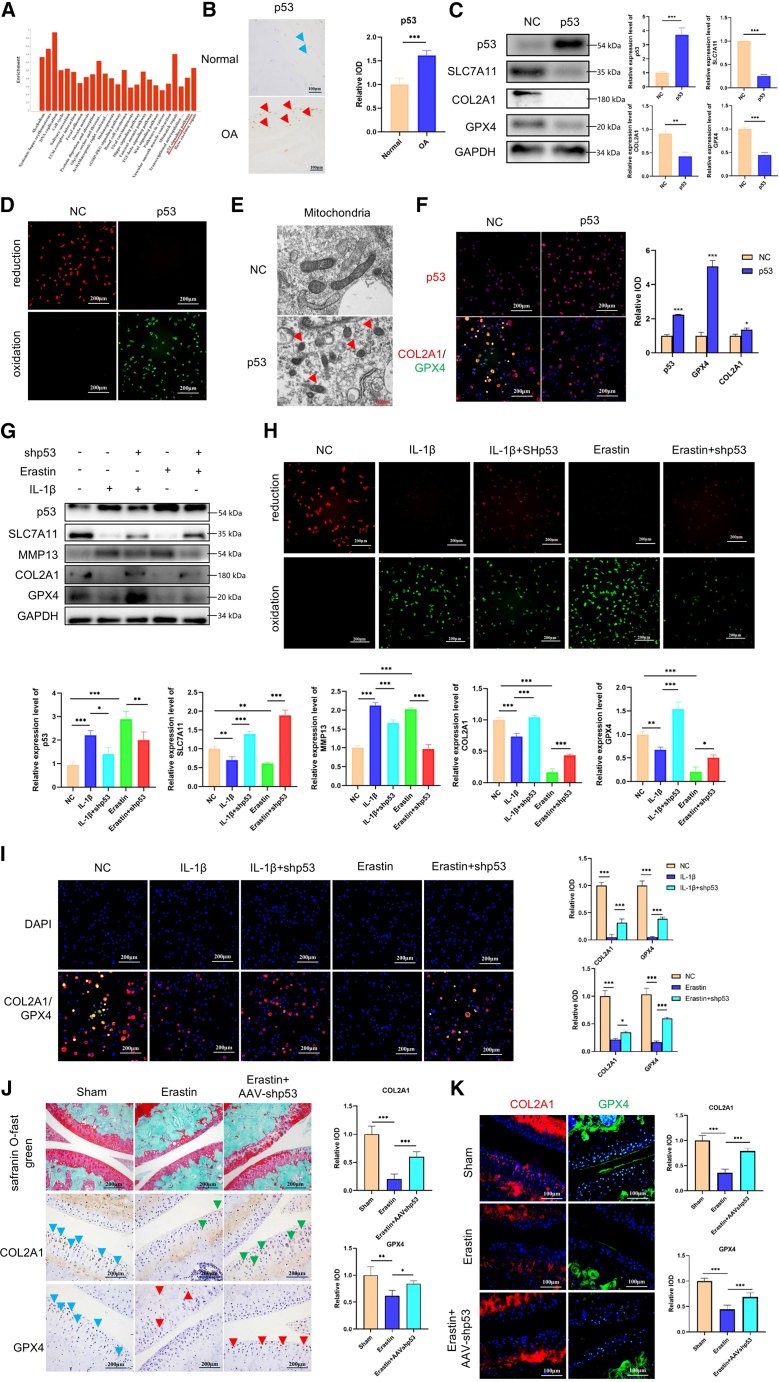
p53 drives ferroptosis and cartilage degeneration in chondrocytes (A) KEGG pathway enrichment analysis of transcriptomic data from OA cartilage, highlighting activation of the p53 signaling pathway. Scale bars, 100 μm. (B) IHC staining showing increased p53 expression in human OA cartilage compared with normal cartilage. (C) Western blot analysis of chondrocytes transfected with p53 overexpression plasmid, showing decreased expression of GPX4, SLC7A11, and COL2A1 levels (unpaired Student’s *t* test). (D) Lipid peroxidation levels in p53-overexpressing chondrocytes. Scale bars, 200 μm. (E) Transmission electron microscopy images showing mitochondrial shrinkage, increased membrane density, and reduced cristae in p53-overexpressing chondrocytes. (F) Immunofluorescence (IF) staining of COL2A1 and GPX4 in chondrocytes with p53 overexpression. Scale bars, 200 μm (unpaired Student’s *t* test). (G) Western blot analysis of chondrocytes treated with erastin with or without p53 knockdown. (H) Lipid peroxidation levels following p53 knockdown under erastin treatment. Scale bars, 200 μm. (I) IF staining showing restoration of COL2A1 and GPX4 expression upon p53 knockdown. Scale bars, 200 μm. (J) Safranin-O/fast green staining of mouse knee joints following intra-articular injection of AAV-shp53 with erastin treatment. Scale bars, 200 μm. (K) *In vivo* fluorescence imaging showing target engagement and protein expression changes following p53 knockdown. Scale bars, 100 μm. Data are representative images or expressed as mean ± SD of each group from at least three experiments; unpaired two-tailed Student’s *t* test (B, C, and F) or one-way ANOVA followed by Tukey’s post hoc test (G, I, J, and K). ∗*p* < 0.05, ∗∗*p* < 0.01.

KEGG pathway analysis of OA transcriptomic data identified activation of the p53 pathway (Figure 2A), which was confirmed by elevated p53 expression in human OA cartilage (Figure 2B). Functionally, p53 overexpression induced an OA-like phenotype, suppressing GPX4, SLC7A11, and COL2A1 while increasing lipid peroxidation and mitochondrial damage (Figures 2C–2E). Immunofluorescence further confirmed reduced COL2A1 and GPX4 expression (Figure 2F). These findings indicate that p53 overexpression is sufficient to drive ferroptotic and catabolic changes.

Conversely, p53 knockdown attenuated erastin-induced ferroptosis, restoring GPX4 and COL2A1 expression and reducing lipid peroxidation (Figures 2G–2I). *In vivo*, AAV-mediated p53 silencing alleviated cartilage degeneration, confirming therapeutic potential (Figures 2J and 2K).

Taken together, p53 acts as a central regulator of ferroptosis in OA chondrocytes.

### RORα exacerbates cartilage degradation in osteoarthritis

**Figure 3:**
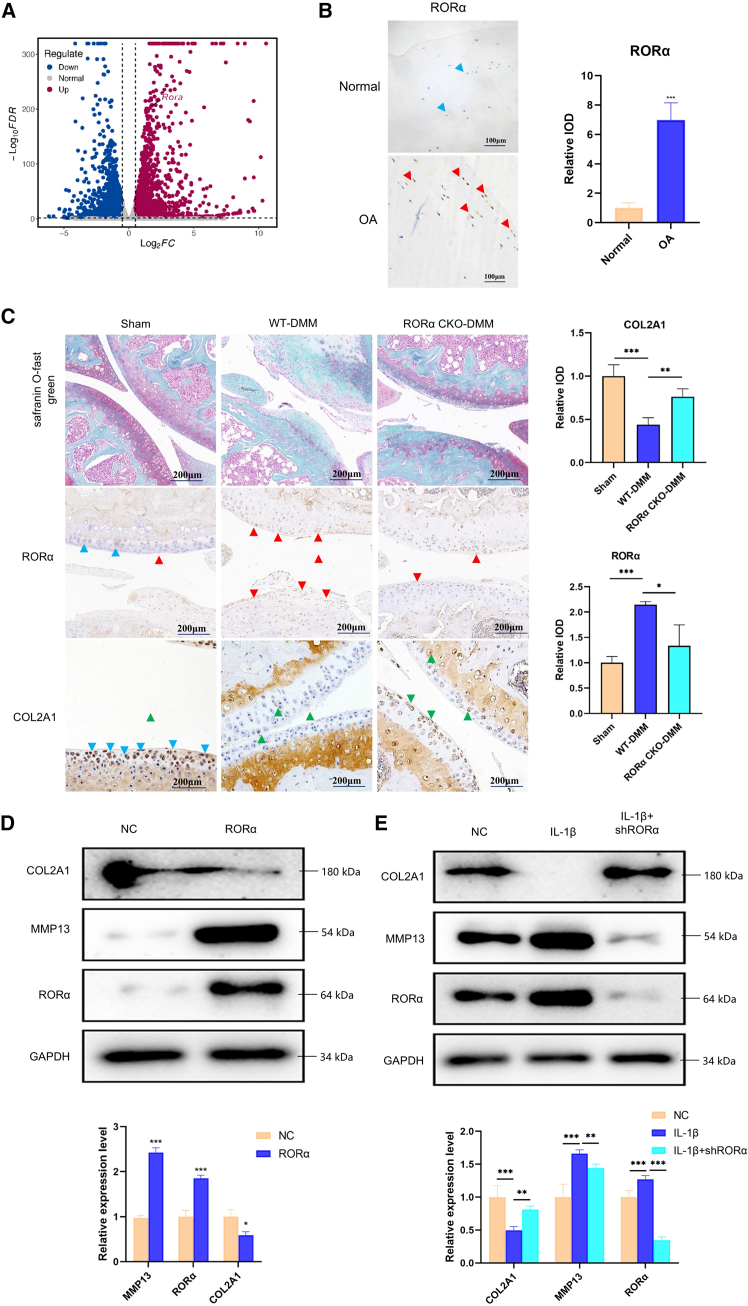
RORα promotes cartilage degradation in osteoarthritis (A) Volcano plot of transcriptomic data showing increased RORα expression in OA chondrocytes compared with normal. (B) IHC staining showing elevated RORα expression in human OA cartilage. Scale bars, 100 μm. (C) Safranin-O/fast green staining and IHC of knee joint sections from control and chondrocyte-specific RORα CKO mice following destabilization of the DMM surgery. Scale bars, 200 μm. (D) Western blot analysis of chondrocytes with RORα overexpression, showing decreased COL2A1 and increased MMP13 expression. (E) Western blot analysis of chondrocytes with RORα knockdown, showing restoration of COL2A1 and reduction of MMP13 expression. Data are representative images or expressed as mean ± SD of each group from at least three experiments; unpaired two-tailed Student’s *t* test (B and D) or one-way ANOVA followed by Tukey’s post hoc test (C and E). ∗*p* < 0.05, ∗∗*p* < 0.01, ∗∗∗*p* < 0.001.

Transcriptomic analysis revealed that RORα was significantly upregulated in OA chondrocytes (Figure 3A), which was validated in human cartilage by immunohistochemistry (Figure 3B).

Chondrocyte-specific RORα cKO mice exhibited marked protection against cartilage degeneration following DMM surgery (Figure 3C). *In vitro*, RORα disrupted chondrocyte homeostasis by suppressing COL2A1 and promoting MMP13 expression, whereas its knockdown restored a balanced phenotype (Figures 3D and 3E).

### RORα orchestrates iron dyshomeostasis and mitochondrial dysfunction in osteoarthritic chondrocytes

**Figure 4:**
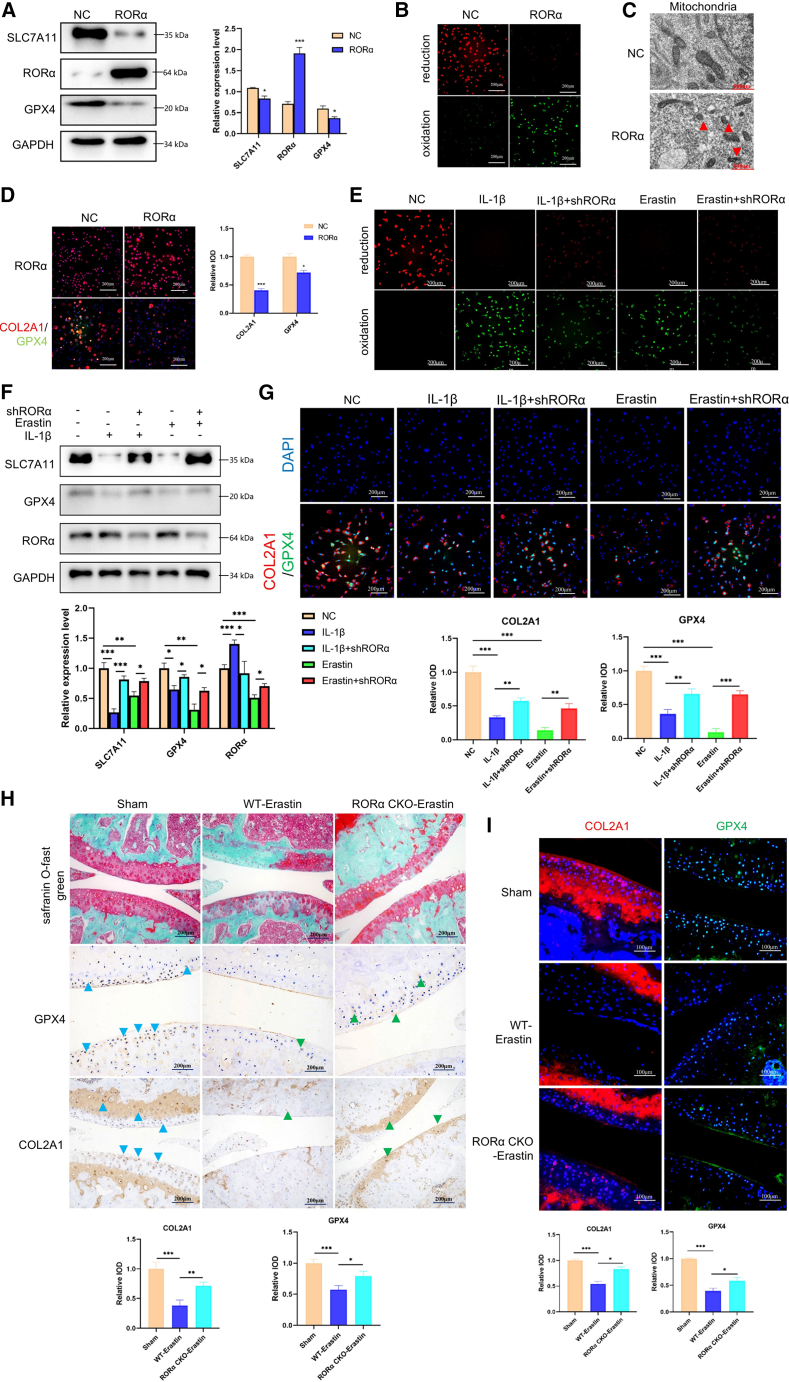
RORα regulates ferroptosis in chondrocytes (A) Western blot analysis of ferroptosis-related proteins in chondrocytes with RORα overexpression. (B) Lipid peroxidation levels in RORα-overexpressing chondrocytes. Scale bars, 200 μm. (C) Transmission electron microscopy images showing mitochondrial shrinkage, increased membrane density, and reduced cristae in RORα-overexpressing chondrocytes. Scale bars, 500 nm. (D) IF staining of COL2A1 and GPX4 in chondrocytes with RORα overexpression. Scale bars, 200 μm. (E) Lipid ROS levels in chondrocytes with RORα knockdown under erastin-induced ferroptotic conditions. Scale bars, 200 μm. (F) Western blot analysis of GPX4 and SLC7A11 proteins in chondrocytes with RORα knockdown under erastin treatment. (G) IF staining showing restoration of COL2A1 and GPX4 expression in RORα knockdown chondrocytes under ferroptotic stress. Scale bars, 200 μm. (H) Safranin-O/fast green and IHC analysis of knee joint cartilage sections from control and chondrocyte-specific RORα CKO mice following erastin administration. Scale bars, 200 μm. (I) IF staining of cartilage tissues showing preserved COL2A1 expression in RORα CKO mice under erastin-induced conditions. Scale bars, 100 μm. Data are representative images or expressed as mean ± SD of each group from at least three experiments; unpaired two-tailed Student’s *t* test (A and D) or one-way ANOVA followed by Tukey’s post hoc test (F, G, H, and I). ∗*p* < 0.05, ∗∗*p* < 0.01, ∗∗∗*p* < 0.001.

RORα gain of function suppressed ferroptosis defense systems, as evidenced by reduced GPX4 and SLC7A11 expression (Figure 4A) and increased lipid ROS (Figure 4B). Transmission electron microscopy (TEM) analysis revealed characteristic ferroptotic mitochondrial damage (Figure 4C), while immunofluorescence confirmed reduced COL2A1 and GPX4 levels (Figure 4D). These findings indicate that RORα activation induces ferroptotic alterations.

Conversely, under ferroptotic stress conditions induced by erastin, RORα knockdown restored GPX4 and SLC7A11 expression, reduced lipid peroxidation, and improved OA-associated markers (Figures 4E–4G), indicating that RORα is required for maintaining ferroptotic signaling.

*In vivo*, RORα CKO mice were protected from erastin-induced cartilage damage, with preserved structure and restored COL2A1 expression (Figures 4H and 4I).

### RORα orchestrates p53-dependent ferroptosis

Rescue experiments demonstrated that p53 knockdown under RORα overexpression markedly suppressed ferroptotic alterations, restoring antioxidant capacity and cartilage anabolism.

**Figure 5:**
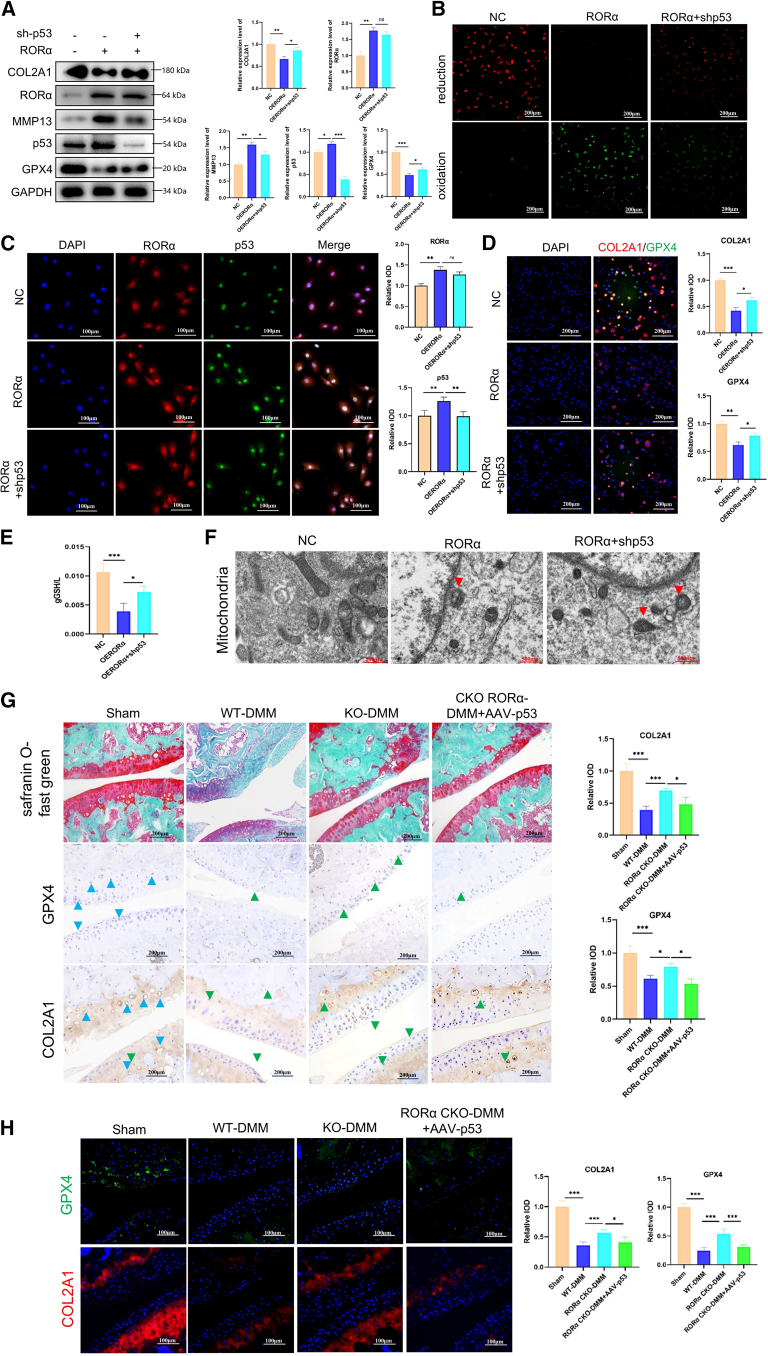
p53 is required for RORα-induced ferroptosis and cartilage degeneration (A) Western blot showing significant upregulation of SLC7A11, GPX4, and COL2A1 expression, alongside downregulation of MMP13 levels following p53 knockdown in RORα-overexpressing chondrocytes. (B) BODIPY 581/591 C11 staining showing decreased lipid peroxidation following p53 knockdown in RORα-overexpressing chondrocytes. Scale bars, 200 μm. (C) IF showing increased expression of p53 following RORα overexpression in chondrocytes. Scale bars, 100 μm. (D) IF showing restoration of COL2A1 and GPX4 expression following p53 knockdown in RORα-overexpressing chondrocytes. Scale bars, 200 μm. (E) Intracellular GSH levels showing recovery of antioxidant capacity following p53 knockdown in RORα-overexpressing chondrocytes. (F) Transmission electron microscopy images showing restored mitochondrial integrity in RORα-overexpressing chondrocytes following p53 knockdown. Scale bars, 500 nm. (G and H) Safranin-O/fast green staining, IHC (scale bars, 200 μm), and IF staining (scale bars, 100 μm) of mouse knee joint tissue sections showing that p53 overexpression negated the protective effects of RORα knockout, leading to severe cartilage degeneration *in vivo*. Scale bars, 200 μm. Data are representative images or expressed as mean ± SD of each group from at least three experiments; one-way ANOVA followed by Tukey’s post hoc test (A, C, D, E, G, and H). ∗*p* < 0.05, ∗∗*p* < 0.01, ∗∗∗*p* < 0.001.

Specifically, the RORα + shp53 group showed increased GPX4, SLC7A11, and COL2A1, along with reduced MMP13 and lipid peroxidation (Figures 5A and 5B). Immunofluorescence confirmed restoration of COL2A1 and GPX4, while RORα overexpression increased p53 expression (Figures 5A–5D). GSH levels were also elevated following p53 knockdown (Figure 5E), indicating recovery of redox balance. TEM analysis showed improved mitochondrial integrity (Figure 5F). *In vivo*, p53 overexpression abolished the protective effect of RORα deficiency, leading to severe cartilage degeneration (Figures 5G and 5H).

These results demonstrate that p53 is required for RORα-induced ferroptosis and cartilage damage.

### The RORα-p53-HAUSP axis regulates p53 ubiquitination

**Figure 6:**
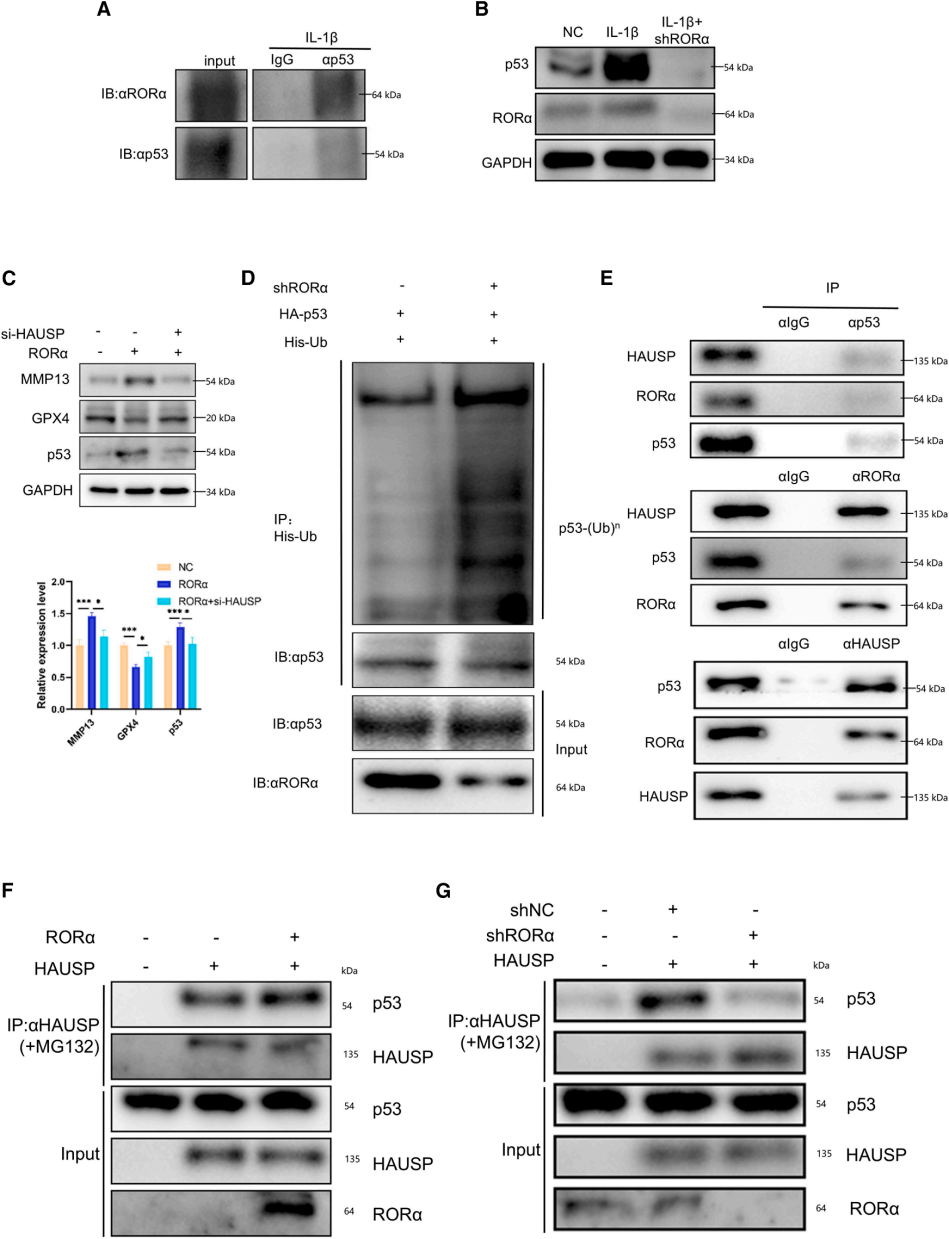
RORα promotes p53 stabilization through HAUSP-dependent deubiquitination (A) CoIP analysis showing the interaction between RORα and p53 in chondrocytes, which is enhanced under IL-1β stimulation. (B) Western blot analysis showing decreased p53 protein levels following RORα knockdown under IL-1β stimulation. (C) Western blot analysis showing that HAUSP knockdown in RORα-overexpressing chondrocytes reduces p53 and MMP13 expression while restoring GPX4 levels. (D) Ubiquitination assay showing increased p53 ubiquitination following RORα depletion. (E) CoIP analysis demonstrating the formation of an RORα-p53-HAUSP complex in chondrocytes. (F) CoIP analysis showing that RORα overexpression enhances the interaction between HAUSP and p53. (G) CoIP analysis showing that RORα inhibition disrupts the HAUSP-p53 interaction. Data are representative images or expressed as mean ± SD of each group from at least three experiments; one-way ANOVA followed by Tukey’s post hoc test (C). ∗*p* < 0.05, ∗∗∗*p* < 0.001.

Co-immunoprecipitation (Co-IP) confirmed an interaction between RORα and p53, which was enhanced by IL-1β stimulation (Figure 6A). RORα knockdown under IL-1β stimulation reduced p53 protein levels, indicating post-translational regulation (Figure 6B).

Importantly, HAUSP knockdown in RORα-overexpressing cells reduced p53 and MMP13 levels while restoring GPX4 expression (Figure 6C), demonstrating that HAUSP is required for RORα-mediated effects. Ubiquitination assays showed that RORα depletion increased p53 ubiquitination, promoting its degradation (Figure 6D). Further analysis revealed that RORα forms a complex with p53 and HAUSP (Figure 6E) and enhances HAUSP-p53 interaction, whereas RORα inhibition disrupts this binding (Figures 6F and 6G). Together, these data indicate that RORα functions as a scaffold to recruit HAUSP, promoting p53 deubiquitination and stabilization, thereby driving ferroptosis.

## Discussion

OA, a heterogeneous degenerative joint disorder, encompasses multiple subtypes with distinct clinical phenotypes driven by complex etiological factors including chronic inflammation, metabolic dysregulation, and programmed cell death modalities. Emerging epidemiological evidence implicates heavy-metal-induced bone homeostasis disruption in degenerative skeletal pathologies such as osteoporosis, OA, and intervertebral disc degeneration. Notably, iron metabolism imbalance has emerged as a pivotal contributor to OA progression. Our multi-model investigation integrating human OA cartilage, *in vitro* systems, and transgenic mice mechanistically establishes ferroptosis as a critical driver of chondrocyte dysfunction and OA pathogenesis.

Among the regulators of ferroptosis, p53 emerges as a central mediator linking oxidative stress to cartilage degeneration. While p53 is classically known to regulate cell-cycle arrest, apoptosis, and senescence, its role in ferroptosis has primarily been characterized in cancer cells. Here, we demonstrate that p53 is markedly upregulated in OA chondrocytes and is required for ferroptosis induction and cartilage degeneration. Genetic silencing of p53 attenuated ferroptotic damage and mitigated OA progression *in vivo*, establishing p53 as a key effector bridging ferroptosis and cartilage pathology.

A major finding of this study is the identification of RORα as an upstream regulator of p53-dependent ferroptosis. RORα, a member of the nuclear receptor family, has been widely implicated in skeletal development, circadian regulation, and lipid metabolism.

Previous studies have shown that RORα contributes to OA progression through the CH25H-CYP7B1-RORα axis by regulating cholesterol metabolism and matrix catabolism. Notably, pharmacological intervention using the RORα-specific inhibitor SR3335 effectively blocks cholesterol-metabolite-mediated aberrant MMP activation, significantly mitigating articular cartilage destruction in surgically induced OA mouse models. In contrast, our findings uncover a distinct mechanism by which RORα promotes OA progression independent of its canonical transcriptional function. We demonstrate that RORα functions as a scaffold protein that recruits the deubiquitinase HAUSP to stabilize p53. This scaffold-mediated mechanism inhibits p53 ubiquitination and proteasomal degradation, thereby amplifying ferroptotic signaling.

Importantly, the transcriptional and scaffold functions of RORα are not mutually exclusive. The cholesterol metabolism axis primarily influences extracellular matrix remodeling and inflammatory responses, whereas the RORα-HAUSP-p53 axis directly governs chondrocyte survival through ferroptosis.^39^^,^^40^^,^^41^ These findings suggest that RORα may act as a context-dependent signaling hub, integrating metabolic and stress-responsive pathways in OA pathogenesis.

In conclusion, this study establishes a RORα-HAUSP-p53 signaling axis that drives ferroptosis and cartilage degeneration, providing a new mechanistic framework and potential therapeutic targets for osteoarthritis.

### Limitations of the study

Despite these findings, several limitations should be acknowledged. First, human samples were obtained from patients undergoing total knee arthroplasty, representing end-stage OA, which limits assessment of disease-stage-specific dynamics. Second, although our data support a functional role of HAUSP in mediating RORα-dependent p53 stabilization, further genetic models (e.g., HAUSP knockout) are required to fully validate its necessity *in vivo*. Third, experimental parameters in the DMM model, including drug dosing and intervention timing, require further standardization to enhance reproducibility. Future studies addressing these limitations will strengthen the mechanistic and translational significance of this pathway.

## Resource availability

### Lead contact

Further information and requests for resources and reagents should be directed to and will be fulfilled by the lead contact, Lei He (helei26@mail.sysu.edu.cn).

### Materials availability

This study did not generate new unique reagents.

### Data and code availability

• Transcriptomic data have been deposited at Mendeley Data and are publicly available as of the date of publication. The dataset is available at Mendeley Data: https://doi.org/10.17632/ymvfj7dg6s.2 and is listed in the key resources table.
• All data reported in this study will be shared by the lead contact upon request.
• This study did not generate new code.
• Any additional information required to reanalyze the data reported in this study is available from the lead contact upon request.

## Acknowledgments

This study was supported by the 10.13039/501100001809National Natural Science Foundation of China (no. 82102576 to L.H. and no. 82202805 to M.Y.), the 10.13039/501100003453Natural Science Foundation of Guangdong Province (no. 2025A1515011091 to L.H.), and the Science and Technology Program of Meizhou (no. 2024C0302004 to L.H.).

## Author contributions

We declare that all the listed authors have participated actively in the study and all meet the requirements of the authorship. L.H., J.D., and M.Y. designed the study and wrote the protocol; L.M., R.Z., and Y.L. acquired the data; J.F. and M.H. analyzed the data; L.M., R.Z., and Y.L. wrote the first draft of the manuscript and mainly revised the manuscript. All authors approved the final version of the manuscript.

## Declaration of interests

The authors declare no competing interests.

## Declaration of generative AI and AI-assisted technologies in the writing process

During the preparation of this work, the author used ChatGPT 5.3 in the writing process in order to improve the readability and language of the manuscript. After using this tool, the author has reviewed and edited the content as needed and takes full responsibility for the content of the publication.

## STAR★Methods

### Key resources table

**Table:** 

REAGENT or RESOURCE	SOURCE	IDENTIFIER
**Antibodies**		

Collagen type II antibody	Abcam	Cat# ab34712, RRID:AB_731688
GPX4 antibody	Proteintech	Cat# 67763-1-Ig, RRID:AB_2909469
MMP13 antibody	Abcam	Cat# ab39012, RRID:AB_776416
RORα antibody	Servicebio	Cat# GB121300
RORα antibody	Abcam	Cat# ab256799, RRID:AB_3697580
ACSL4 antibody	Abcam	Cat# ab155282, RRID:AB_2714020
p53 antibody	Abcam	Cat# ab26, RRID:AB_303198
HAUSP antibody	Abcam	Cat# ab264422
Ubiquitin antibody	Abcam	Cat# ab134953, RRID:AB_2801561
SLC7A11 antibody	Abcam	Cat# ab307601, RRID:AB_3094570
Secondary antibody (biotinylated)	Thermo Fisher Scientific	Cat# 31820, RRID:AB_228340

**Biological samples**		

Human knee cartilage (OA and normal paired samples, *n* = 12)	Human knee cartilage (OA and normal paired samples, *n* = 12)	Human knee cartilage (OA and normal paired samples, *n* = 12)

**Chemicals, peptides, and recombinant proteins**		

Deferoxamine (DFO)	Sigma-Aldrich	N/A
Erastin	Selleck Chemicals	S7243
BODIPY 581/591 C11	Invitrogen	D3861
Triton X-100	Sigma-Aldrich/BIOFROXX	1139ML100
DAPI	Beyotime	C1005
Paraformaldehyde	Servicebio	N/A
Trypsin (0.25%)	Gibco	N/A
Type II collagenase	Gibco/Sigma	N/A
RIPA buffer	Beyotime	P0013B
EDTA (20%)	Standard reagent	N/A
PBS	Biosharp	BL302A

**Critical commercial assays**		

GSH detection kit	Beyotime	S0053
Total Iron Assay Kit	Nanjing Jiancheng	A039-2-1
NEBNext Ultra RNA Library Prep Kit	NEB	E7530
TRIzol reagent	Invitrogen	15596026

**Deposited data**		

Transcriptomic data	This paper	Mendeley Data: https://doi.org/10.17632/ymvfj7dg6s.2

**Experimental models: Cell lines**		

DMEM/F12 medium	Gibco	N/A
Fetal bovine serum (FBS)	Gibco	N/A
Polybrene	Beyotime	C0351

**Experimental models: Organisms/strains**		

RORα flox/flox mice	This paper	N/A
Col2a1-creERT2 mice	This paper	N/A
RORα conditional knockout (Col2a1-creERT2+/−; RORα flox/flox)	This paper	N/A
C57BL/6 mice	C57BL/6 mice	C57BL/6 mice

**Oligonucleotides**		

RORα overexpression lentivirus	Cyagen	[pLV-EF1a>mRora/3xFLAG-CMV>eGFP/T2A/Puro]
Empty-vector control lentivirus	Cyagen	[pLV-CMV>eGFP/T2A/Puro]
RORα shRNA lentivirus	Cyagen	shRNA sequence: 5′-GAATCCAT TATGGTGTCATTACTCGAGTAAT GACACCATAATGGATTC-3′
Non-targeting shRNA control lentivirus	Cyagen	shNC sequence: 5′-cctaaggttaagtcgcc ctcgctcgagcgagggcgacttaaccttagg-3′

**Software and algorithms**		

Hitachi TEM system	Hitachi	Hitachi
Olympus BX51 microscope	Olympus	N/A
BIO-RAD ChemiDoc XRS	Bio-Rad	N/A
NanoDrop 2000	Thermo Fisher	N/A
Agilent 2100 Bioanalyzer	Agilent	N/A
Illumina NovaSeq 6000	Illumina	N/A
Hitachi TEM system	Hitachi	Hitachi

### Experimental model and study participant details

#### Human samples

Joint samples were collected from 12 patients undergoing unilateral total knee arthroplasty (TKA) for primary knee osteoarthritis. Specimens, including full-thickness articular cartilage and subchondral bone, were obtained during surgery and divided into macroscopically damaged regions (OA group) and relatively preserved regions (normal group) within the same joint. This paired-sampling strategy minimized inter-individual variability under identical systemic and mechanical conditions.

Clinical characteristics including age, sex, affected side, and disease duration were collected for all enrolled patients to minimize sampling bias. All patients were diagnosed with end-stage primary knee osteoarthritis (Kellgren–Lawrence grade IV) and underwent unilateral TKA. Patients with inflammatory arthritis, metabolic bone disease, malignancy, or a history of prior knee surgery were excluded (Table S1).

All human studies were approved by the Ethics Committee of the Third Affiliated Hospital of Sun Yat-sen University (Approval No. RG2023-036-01). The requirement for written informed consent was waived by the Ethics Committee due to the use of anonymized, archived human specimens.

The inclusion of TKA-derived cartilage samples was intentionally designed to obtain sufficient and structurally intact tissue for mechanistic analyses of ferroptosis and protein–protein interaction networks.

#### Animals and experimental design

This study was approved by the ethical committee of the third affiliated hospital of Sun Yat-sen University (Guangzhou, China) and have been performed in line with the principles of the Declaration of Helsinki. All animal experiments were approved by the Experimental Animal Ethics Committee of South China Agricultural University ([No. 2021F107], Guangzhou, China) and were performed in accordance with the guidelines and regulations of the committee.

To generate chondrocyte-specific RORα knockout mice, RORα flox/flox mice (homozygous for loxP-flanked RORα alleles) were crossed with Col2a1-creERT2 transgenic mice (expressing tamoxifen-inducible Cre recombinase under the collagen type II alpha 1 promoter). The final homozygous mice (experimental group: Col2a1-creERT2+/−; RORα flox/flox) are generated through a multi-step breeding and genotyping strategy (Table S2).

RORα conditional knockout mouse model (RORα CKO mouse): 10 mL of corn oil was sterilized (autoclaved at 121°C for 30 min, cooled to room temperature). 100 mg of Tamoxifen (TargetMol, catalog number: T6906) was weighed and dissolved in 0.5 mL of anhydrous ethanol at 55°C for 1 h. After complete dissolution, 9.5 mL of corn oil was added and mixed for 30 min on a rotator to obtain a 10 mg/mL Tamoxifen solution. Mice were intraperitoneally injected with 10 mg/mL Tamoxifen at a dose of 75 mg/kg for 7 consecutive days, with one injection per day. After the treatment, mice were maintained for an additional 7 days before proceeding with subsequent experiments.

Eight-week-old male wild-type C57BL/6 mice underwent destabilization of the medial meniscus (DMM) surgery on the right knee to induce knee joint instability and establish an OA animal model. Mice were anesthetized with an intraperitoneal injection of 0.5% pentobarbital. After shaving and strict disinfection of the surgical area, mice were fixed in a supine position. The skin of the right knee was incised, the joint capsule was opened, the medial collateral ligament of the medial meniscus was transected, and then the joint cavity and skin incisions were sutured. The sham surgery group only had the right knee joint capsule incised and sutured. We randomly divided mice into 8 groups with 9 mice per group: Sham, DMM, DMM + DFO, Erastin, Erastin + AAV-shp53, RORα CKO- DMM, RORα CKO- Erastin, RORα CKO- DMM + AAV-p53. For drug interventions, deferoxamine (DFO, 100 mg/kg) was administered via intraperitoneal injection daily starting one week after DMM surgery until sacrifice at 8 weeks post-surgery. Erastin (20 mg/kg) or vehicle was administered via intra-articular injection twice a week for 4 weeks. These doses and regimens were selected based on preliminary experiments and previous literature demonstrating efficacy in modulating iron metabolism or inducing ferroptosis in joint tissues.

All animal experiments were approved by the Experimental Animal Ethics Committee of South China Agricultural University ([No. 2021F107]) and performed in compliance with institutional guidelines for the care and use of laboratory animals.

### Method details

#### Isolation and culture of chondrocytes

Primary mouse chondrocytes were isolated from the femoral head, femoral condyles, and tibial plateau of 3-day-old C57BL/6 mouse pups. The process involved cutting the articular cartilage into small pieces and digesting them at 37°C with 0.25% trypsin for 30 min. After washing three times with PBS (Biosharp, catalog number: BL302A), the cartilage was fully digested with 2% type II collagenase at 37°C for 24 h. The cell suspension was then filtered through a 70 μm cell strainer and centrifuged at 1000 rpm for 5 min to collect primary chondrocytes. The cells were cultured in DMEM/F12 medium (Gibco) containing 5% fetal bovine serum (FBS). All cells were maintained in a humidified incubator at 37°C with 5% CO2.

#### Overexpression/knockdown of target genes in chondrocytes

To specifically overexpress or knock down target genes, Lentivirus carrying OE RNA/SH RNA constructs (multiplicity of infection [MOI] = 50) was used to transduce 1 × 10ˆ6 cells overnight at 37°C in the presence of 5 μg/mL polybrene (Beyotime, catalog number: C0351). Lentivirus packaging was provided by Cyagen.

#### Measurement of glutathione (GSH)

After 48 h of the indicated treatment, GSH levels in cell lysates were quantified using a GSH detection kit (Beyotime, S0053) according to the manufacturer’s protocol. The assay measures absorbance of reaction-specific substrates using a microplate reader (BioTek Synergy H1). Data were normalized to total protein concentration determined by BCA assay.

#### Measurement of total iron levels

Total iron content in cells or tissues was determined using a Total Iron Assay Kit (Nanjing Jiancheng Bioengineering Institute, A039-2-1) following the manufacturer’s protocol. Briefly, samples were homogenized in saline and mixed with acid digestion buffer to release bound iron. After centrifugation (12,000 × g, 10 min), the supernatant was incubated with chromogenic substrate at 37°C for 10 min. Absorbance was measured at 520 nm using a spectrophotometer (Thermo Scientific Multiskan GO). Iron concentration was calculated based on a standard curve and normalized to total protein content (determined by BCA assay).

#### Immunohistochemistry

For immunohistochemical (IHC) staining, sections were heated at 95°C for 15 min and then treated with 3% H2O2 and 0.5% Triton X-100 (BIOFROXX, catalog number: 1139ML100). The sections were blocked with 10% bovine serum albumin at room temperature for 1 h to prevent nonspecific binding. The sections were then incubated with primary antibodies overnight at 4°C. Subsequently, the sections were incubated with biotinylated secondary antibodies (Invitrogen,31820,1:200), counterstained with hematoxylin, and visualized with DAB solution under an upright microscope. Primary antibodies used were: collagen type II (Col2a1) antibody (Abcam, ab34712,1:20); glutathione peroxidase 4 (GPX4) antibody (Proteintech, 67763-1-Ig,1:400); matrix metalloproteinase 13 (MMP13) antibody (Abcam, ab39012,1:200); retinoic acid-related orphan receptor RORα antibody (Servicebio, GB121300,1:800); and long-chain fatty acid-CoA synthetase 4 (ACSL4) antibody (Abcam, ab155282,1:200).

#### Assessment of lipid peroxidation by BODIPY 581/591 C11 staining

Chondrocytes were seeded in 48-well plates and stained with 5 μM BODIPY 581/591 C11 (Invitrogen, catalog number: D3861) at 37°C for 30 min. After washing with HBSS, the cells were immediately observed under a fluorescence microscope. Fluorescence intensity was monitored in the FITC and Texas Red channels.

#### Transmission electron microscopy (TEM) assays

TEM was used to observe mitochondrial morphological changes in chondrocytes. Cells were washed with PBS (Biosharp, catalog number: BL302A) and then fixed with electron microscopy fixative (Servicebio, G1102). The samples were dehydrated with different concentrations of alcohol and acetone. Subsequently, the samples were rinsed with propylene oxide and infiltrated with epoxy resin. Ultrathin sections were stained with 1% uranyl acetate and 0.1% lead citrate and scanned using an 80 kV Hitachi TEM system.

#### Western blot

Chondrocytes were lysed with RIPA lysis buffer (Beyotime, catalog number: P0013B). Proteins were separated by 8% SDS polyacrylamide gel electrophoresis (PAGE) and transferred to polyvinylidene fluoride (PVDF) membranes. The PVDF membranes were incubated with primary antibodies overnight at 4°C and with secondary antibodies for 1 h at room temperature. The proteins were visualized using a BIO-RAD ChemiDoc XRS system. Primary antibodies used were: COL2A1 antibody (Abcam, ab34712, 1:1000); GPX4 antibody (Proteintech, 67763-1-Ig,1:1000); MMP13 antibody (Abcam, ab39012,1:1000); RORα antibody (Abcam, ab256799, 1:1000) and SLC7A11 antibody (Abcam, ab307601,1:1000); P53(Abcam, ab26, 1:1000).

#### Safranin-O staining

Tissues were fixed with 4% buffered paraformaldehyde and then decalcified with buffered EDTA (20% EDTA, pH 7.4). The tissues were embedded in paraffin, and sections were stained with Safranin-*O*-fast green. The cell structure and morphology of cartilage and subchondral bone were examined under an upright microscope in a blinded manner.

#### Immunofluorescence (IF) staining

Chondrocytes were seeded in 24-well culture plates and fixed with 4% paraformaldehyde for 30 min. The cells were then permeabilized with 0.3% Triton X-100 (Sigma-Aldrich, T8787) for 1 h. Cells were incubated with primary antibodies against RORα (Servicebio, GB121300; mouse anti; diluted 1:800), p53 (Servicebio, GB111740; diluted 1:500), Col2a1 (Abcam, ab34712, 1:200), and GPX4 (Proteintech, 67763-1-Ig,1:400), followed by the respective fluorescent secondary antibodies. After incubation, cells were washed and stained with DAPI (Beyotime, C1005, 1:5000). The cells were observed under a fluorescence microscope (OLYMPUS BX51, Japan).

#### Co-immunoprecipitation (Co-IP)

Co-IP was performed to investigate protein-protein interactions using the following antibodies: P53 antibody (Abcam, ab26), HAUSP antibody (Abcam, ab264422), Ubiquitin antibody (Abcam, ab134953), RORα antibody (Abcam, ab256799) and an isotype-matched IgG control (Beyotime, A7028). Cellular lysates were prepared by harvesting cells in ice-cold IP lysis buffer containing protease inhibitors, followed by 20-min incubation on ice. After centrifugation at 14,000 × g for 20 min at 4°C, clarified supernatants were collected and incubated with 10 μg of primary antibodies under constant rotation overnight at 4°C. Protein complexes were then captured by adding 25 μL of pre-equilibrated Protein A/G Magnetic Beads (Beyotime, P2108) and incubating for 2 h at 4°C. Beads were magnetically isolated, washed sequentially with IP buffer and ultrapure water, and bound proteins were eluted in 50 μL Lane Marker Sample Buffer. Samples were denatured at 100°C for 10 min prior to separation by SDS-PAGE and immunoblotting analysis.

#### Transcriptomics analysis (RNA-Seq)

Total RNA was extracted from primary chondrocytes using TRIzol reagent (Invitrogen) according to the manufacturer’s protocol. The quantity and purity of the extracted RNA were assessed using a NanoDrop 2000 spectrophotometer (Thermo Fisher Scientific). RNA integrity was evaluated using the RNA Integrity Number (RIN) determined by an Agilent 2100 Bioanalyzer (Agilent Technologies). Only samples with a RIN >8.0 were used for subsequent library construction.

Sequencing libraries were prepared from 1 μg of total RNA using the NEBNext Ultra RNA Library Prep Kit for Illumina (NEB, USA) following the manufacturer’s instructions. Briefly, messenger RNA (mRNA) was enriched from total RNA using oligo(dT) magnetic beads. The enriched mRNA was then fragmented into short pieces. First-strand cDNA was synthesized using random hexamer primers and M-MuLV Reverse Transcriptase, followed by second-strand cDNA synthesis using DNA Polymerase I and RNase H. The resulting double-stranded cDNA was end-repaired, A-tailed, and ligated with sequencing adapters. The ligated products were purified and enriched by PCR amplification to create the final cDNA library. The quality of the library was assessed on an Agilent Bioanalyzer 2100 system.

The final libraries were sequenced on an Illumina NovaSeq 6000 platform with a paired-end 150 bp (PE150) sequencing strategy.

Raw sequencing reads were first processed to remove adapter sequences and low-quality reads using Trimmomatic v0.39. The quality of the clean reads was verified using FastQC v0.11.9. The clean reads were then aligned to the human reference genome (GRCh38) using HISAT2 v2.2.1. Gene expression levels were quantified as read counts for each gene using featureCounts v2.0.1.

Differential expression analysis between comparison groups was performed using the DESeq2 R package. Genes with an adjusted *p*-value (padj) < 0.05 and an absolute log2(Fold Change) > 1 were considered differentially expressed genes (DEGs). Gene Ontology (GO) and Kyoto Encyclopedia of Genes and Genomes (KEGG) pathway enrichment analyses of the DEGs were performed using the clusterProfiler R package to identify their associated biological functions and pathways.

### Quantification and statistical analysis

All data are presented as mean ± S.D. Statistical analyses were performed using GraphPad Prism. Unpaired Student’s t tests were used for comparisons between two groups. One-way ANOVA followed by Tukey-Kramer multiple comparisons tests was used for comparisons among multiple groups, as appropriate. Statistical significance was defined as *p* < 0.05. In the figures, asterisks indicate statistical significance as follows: ∗*p* < 0.05, ∗∗*p* < 0.01, and ∗∗∗*p* < 0.001.
