# Supplementary material for: Mechanism of RORα in promoting osteoarthritis through p53 deubiquitination-mediated chondrocyte ferroptosis
Source: iScience. 2026 Jul 27;29(8):116939. doi: 10.1016/j.isci.2026.116939 (PMC13446196; doi:10.1016/j.isci.2026.116939)

iScience, Volume *29*

Supplemental information

Mechanism of RORα in promoting osteoarthritis through p53 deubiquitination-mediated chondrocyte ferroptosis

Lang Mai, Ruijue Zhu, Yankui Liu, Jingxuan Feng, Mudan Huang, Mingyu Yin, Jiawei Di, and Lei He

Table S1. Clinical characteristics of patients with osteoarthritis included in this study

Patient ID	Age (years)	Sex	Affected Side	Disease Duration (years)
01	68	Female	Right	8
02	72	Male	Left	10
03	65	Female	Right	6
04	70	Female	Left	12
05	74	Male	Right	9
06	69	Female	Left	7
07	66	Male	Right	5
08	71	Female	Left	11
09	73	Female	Right	10
10	67	Male	Left	6
11	75	Female	Right	13
12	68	Male	Left	8

Table S2. Primers for real-time PCR analyses for genotyping assays

**Name**	**Sequence**	**Product length (bp)**
RORα floxed	F:5’-TTAAGAACGGTAACTTGAGGTGAGTCAC-3’<br>R:5’-CTTGCCCTTGGTCCATAATCATTC-3’	332
Cre transgene	F:5’-GGGCAGTCTGGTACTTCCAAGCT-3’<br>R:5’-ATGAGCCACCATGTGGGTGTC-3’	356

Data S1: The raw blot images.

Fig1 H

COL2A1

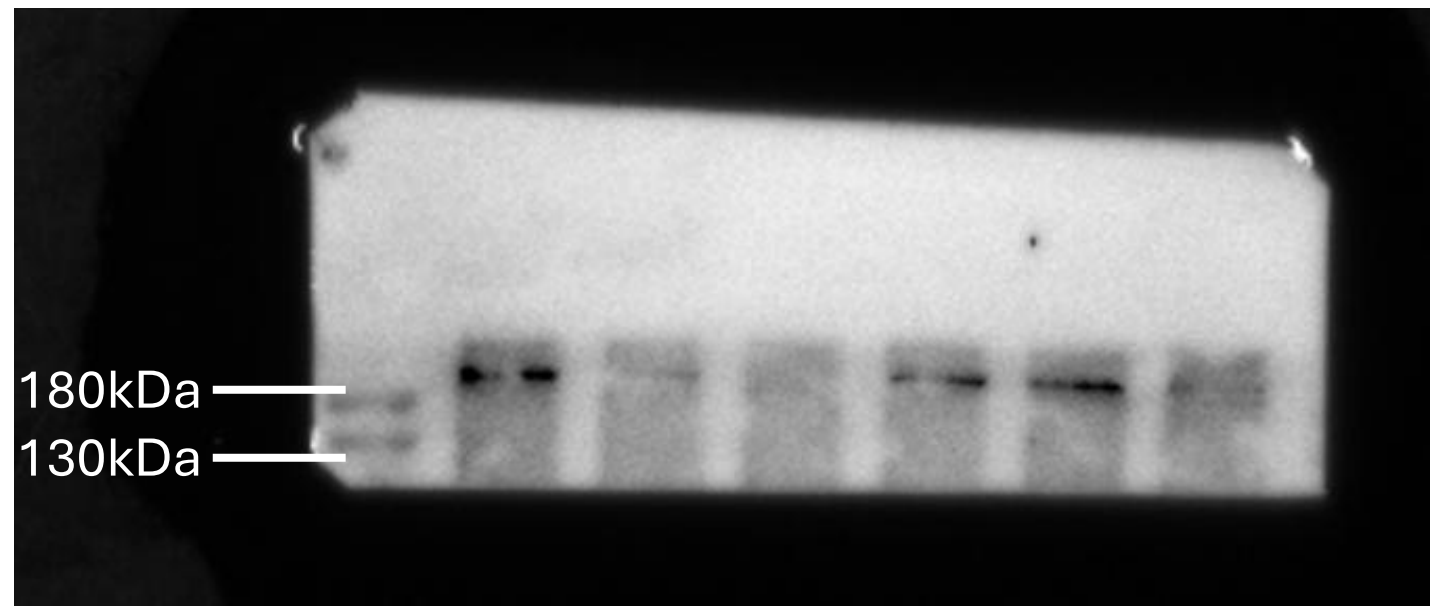

GAPDH

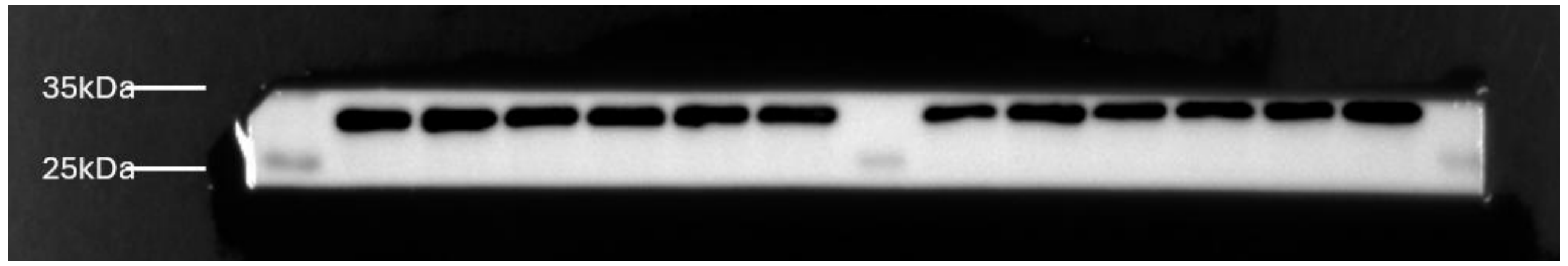

FTH1

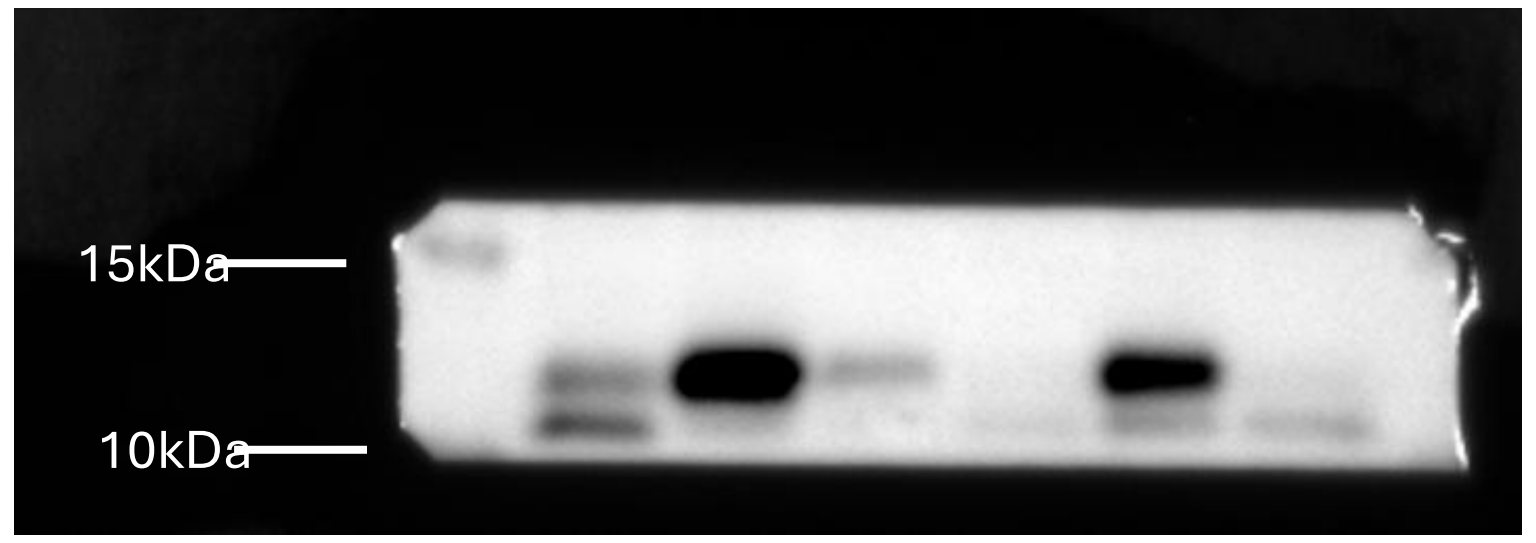

MMP13

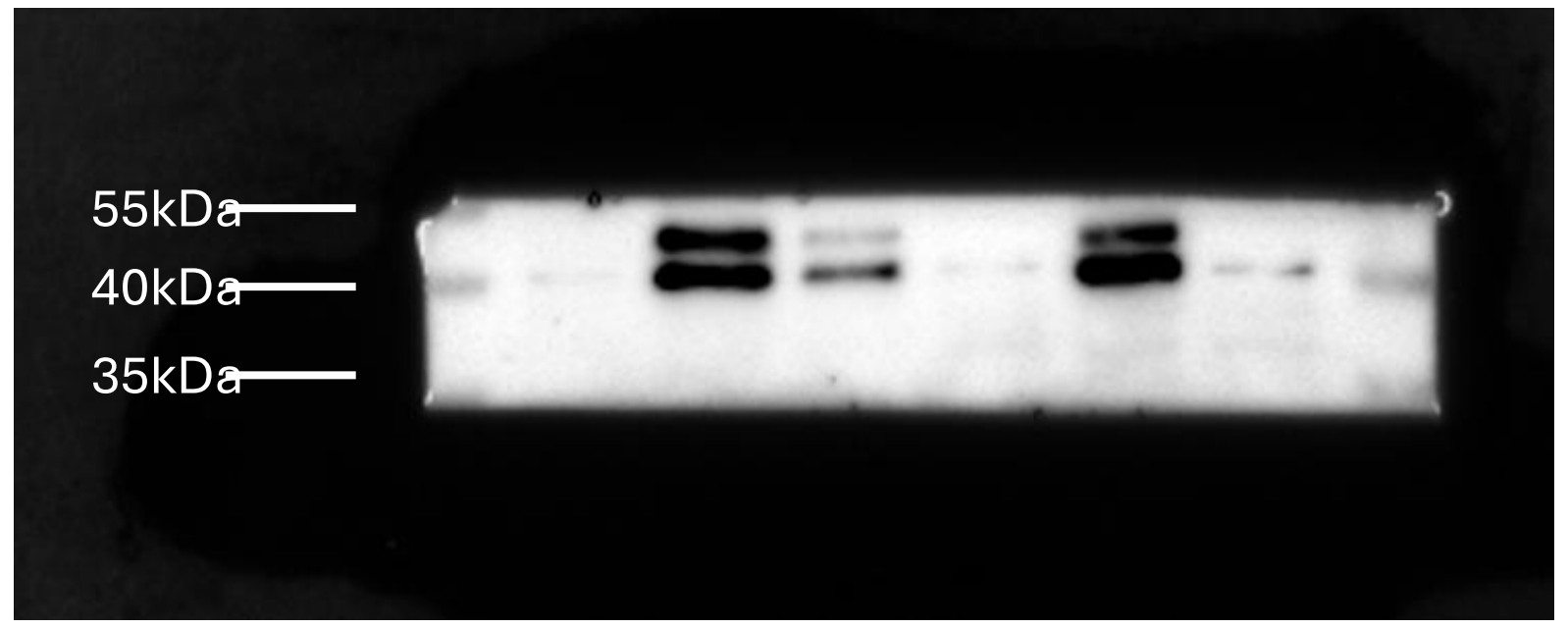

Fig 2C

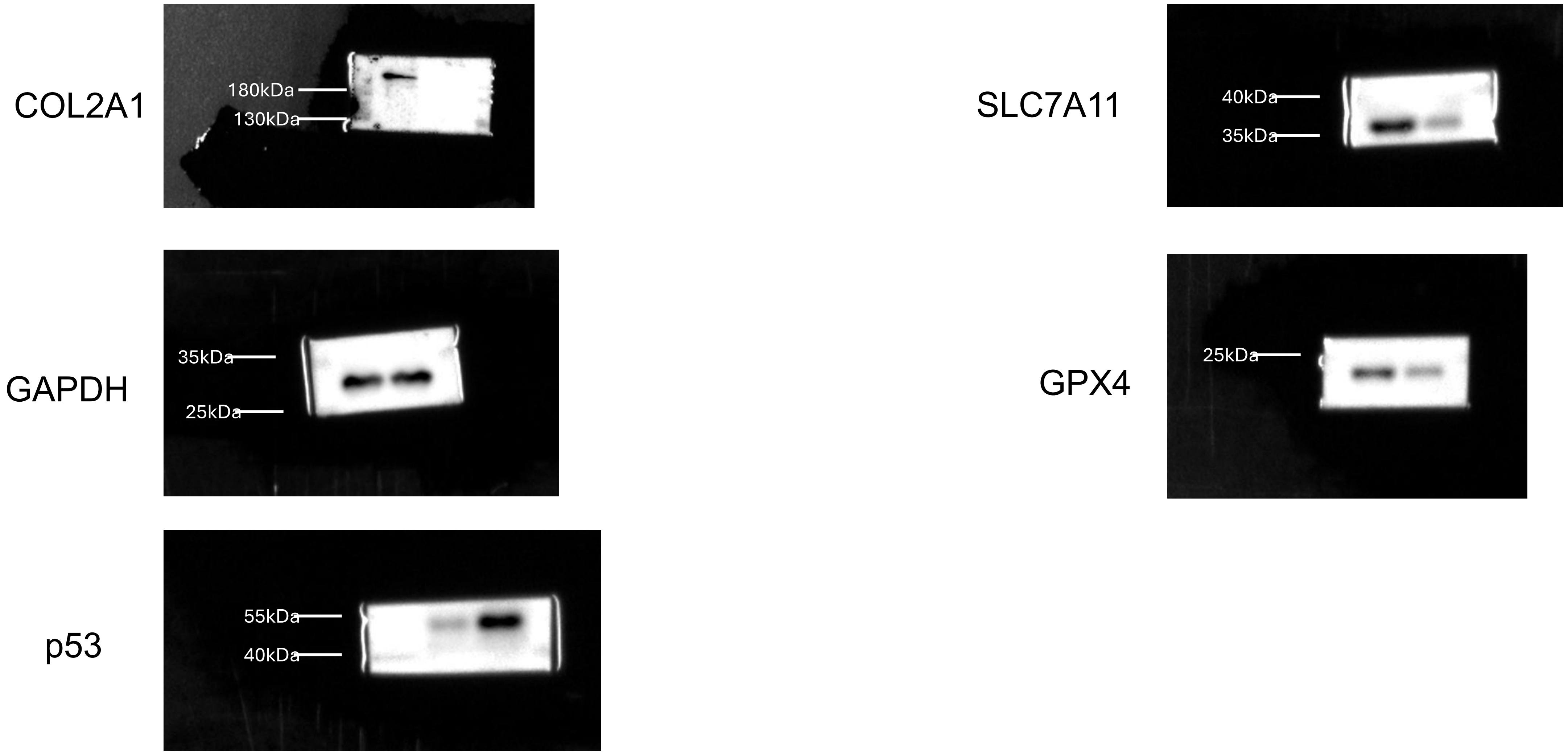
COL2A1
180kDa
130kDa
GAPDH
35kDa
25kDa
p53
55kDa
40kDa
SLC7A11
40kDa
35kDa
GPX4
25kDa

Fig2 G

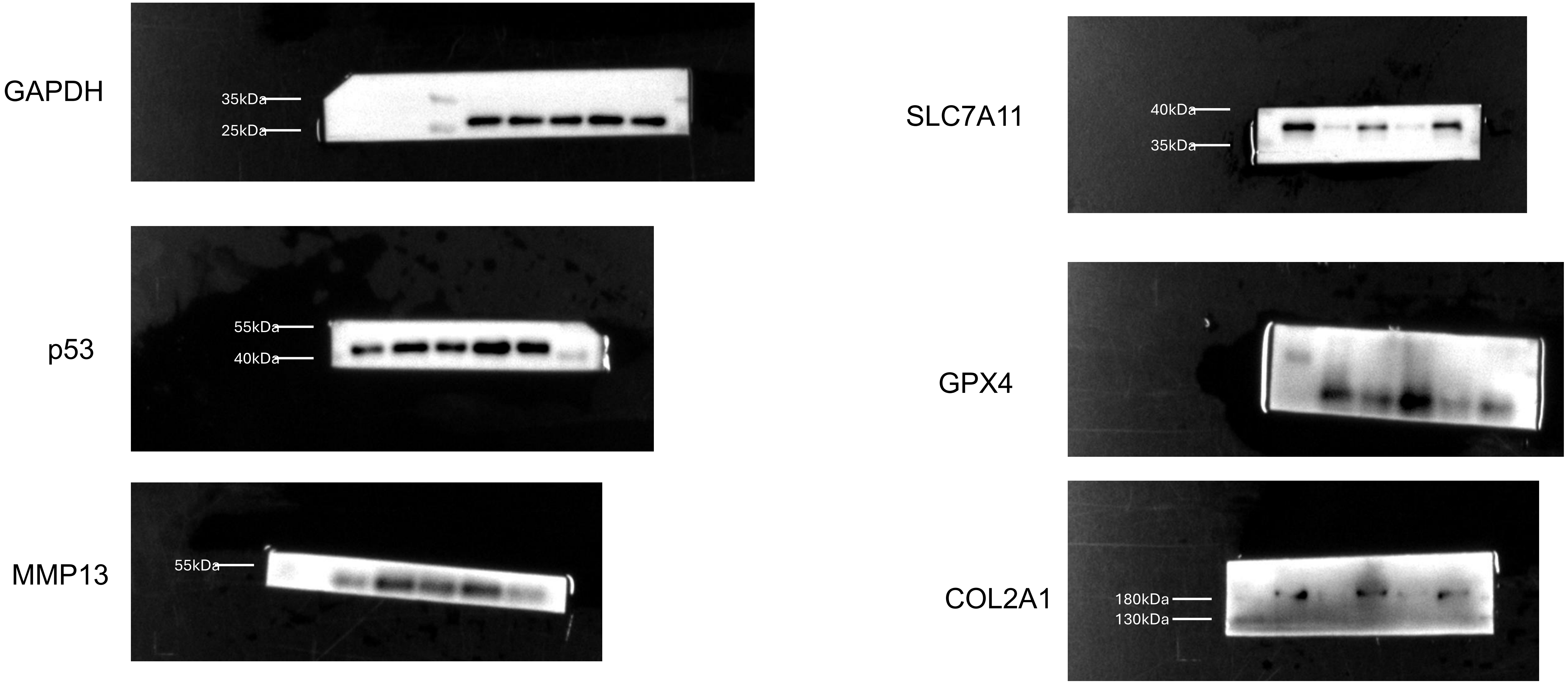
GAPDH
35kDa
25kDa
p53
55kDa
40kDa
MMP13
55kDa
SLC7A11
40kDa
35kDa
GPX4
COL2A1
180kDa
130kDa

Fig3 D

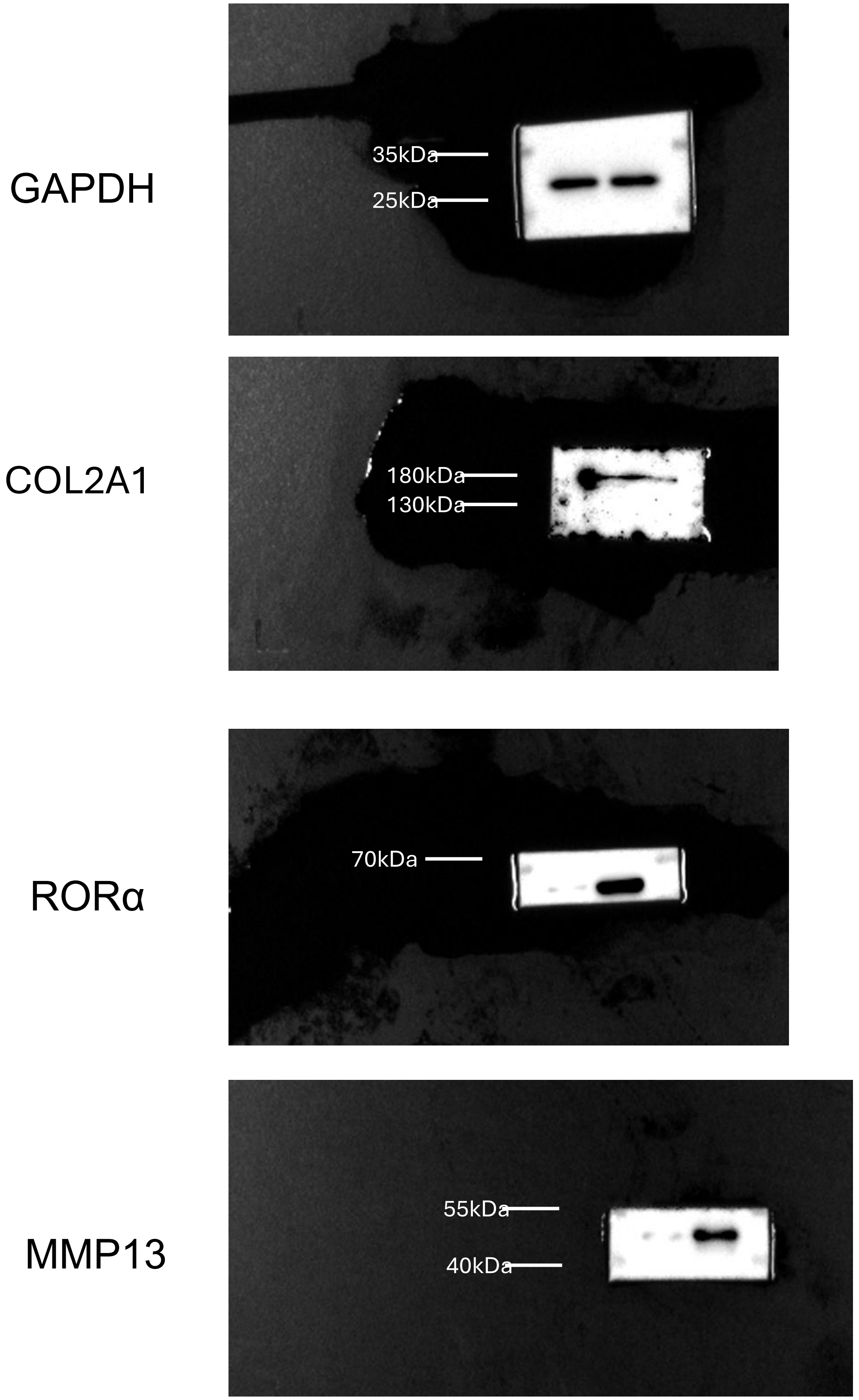
GAPDH
35kDa
25kDa
COL2A1
180kDa
130kDa
RORα
70kDa
MMP13
55kDa
40kDa

Fig3 E

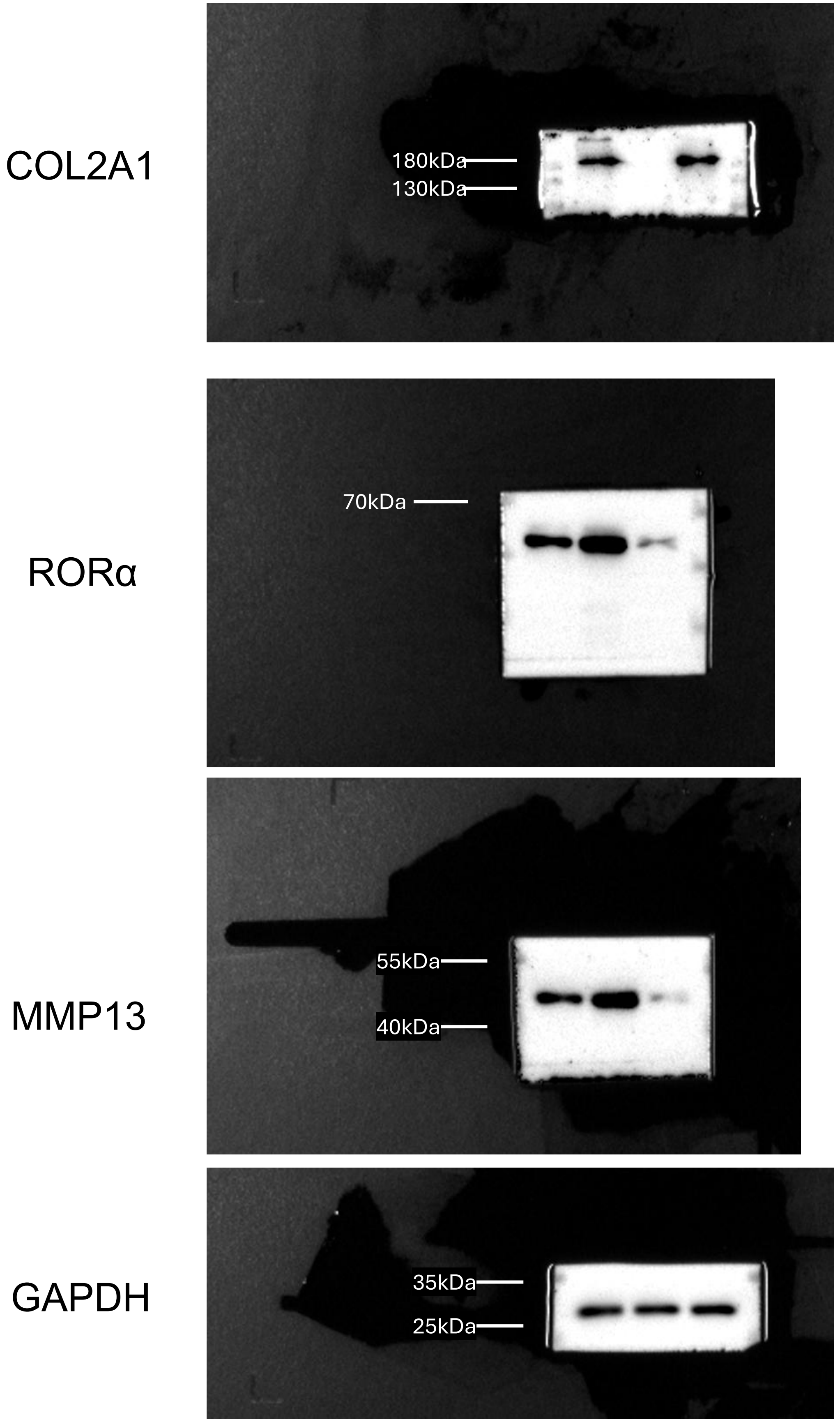

COL2A1
180kDa
130kDa
RORα
70kDa
MMP13
55kDa
40kDa
GAPDH
35kDa
25kDa

Fig4 A

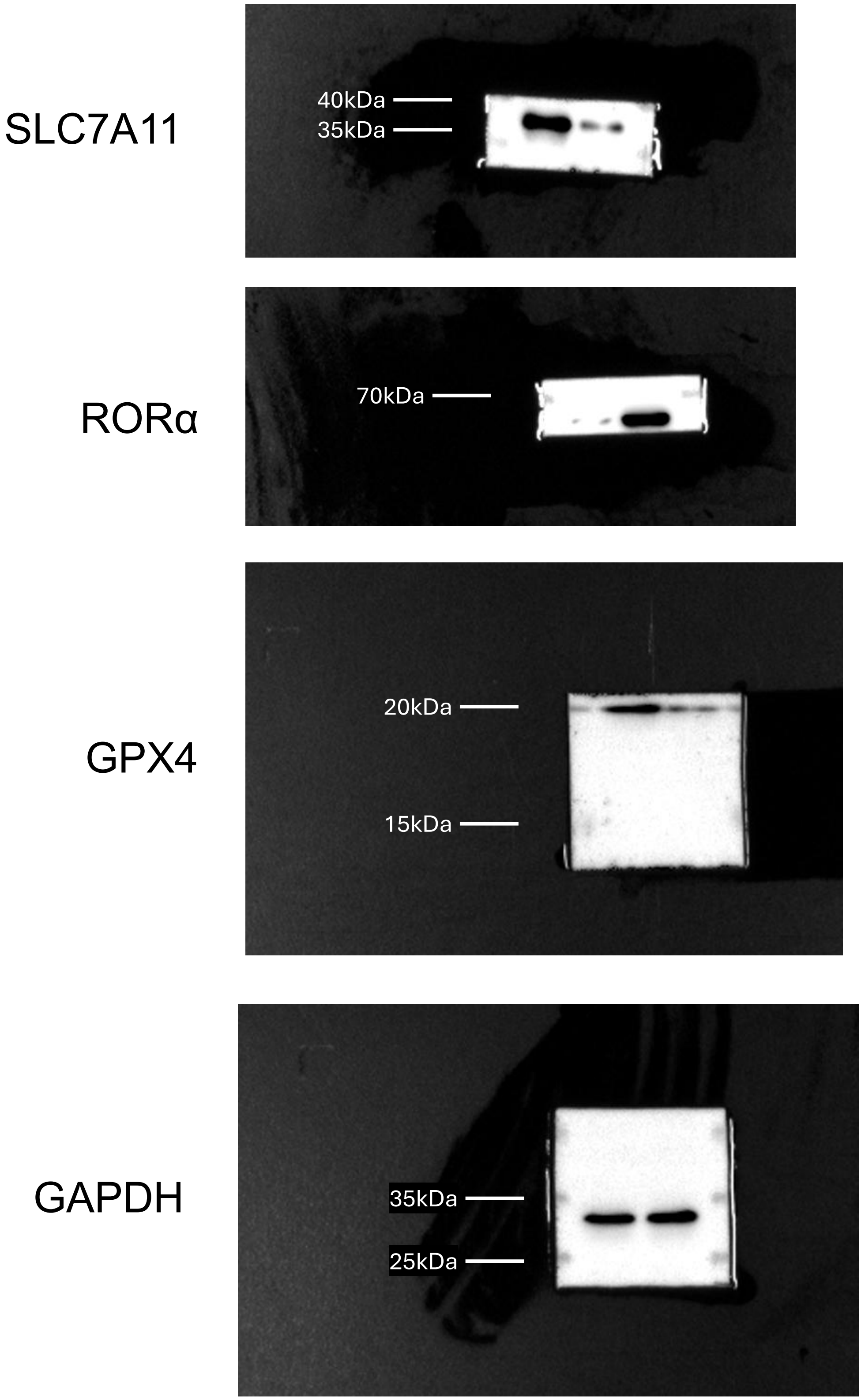
SLC7A11
40kDa
35kDa
RORα
70kDa
GPX4
20kDa
15kDa
GAPDH
35kDa
25kDa

Fig4 F

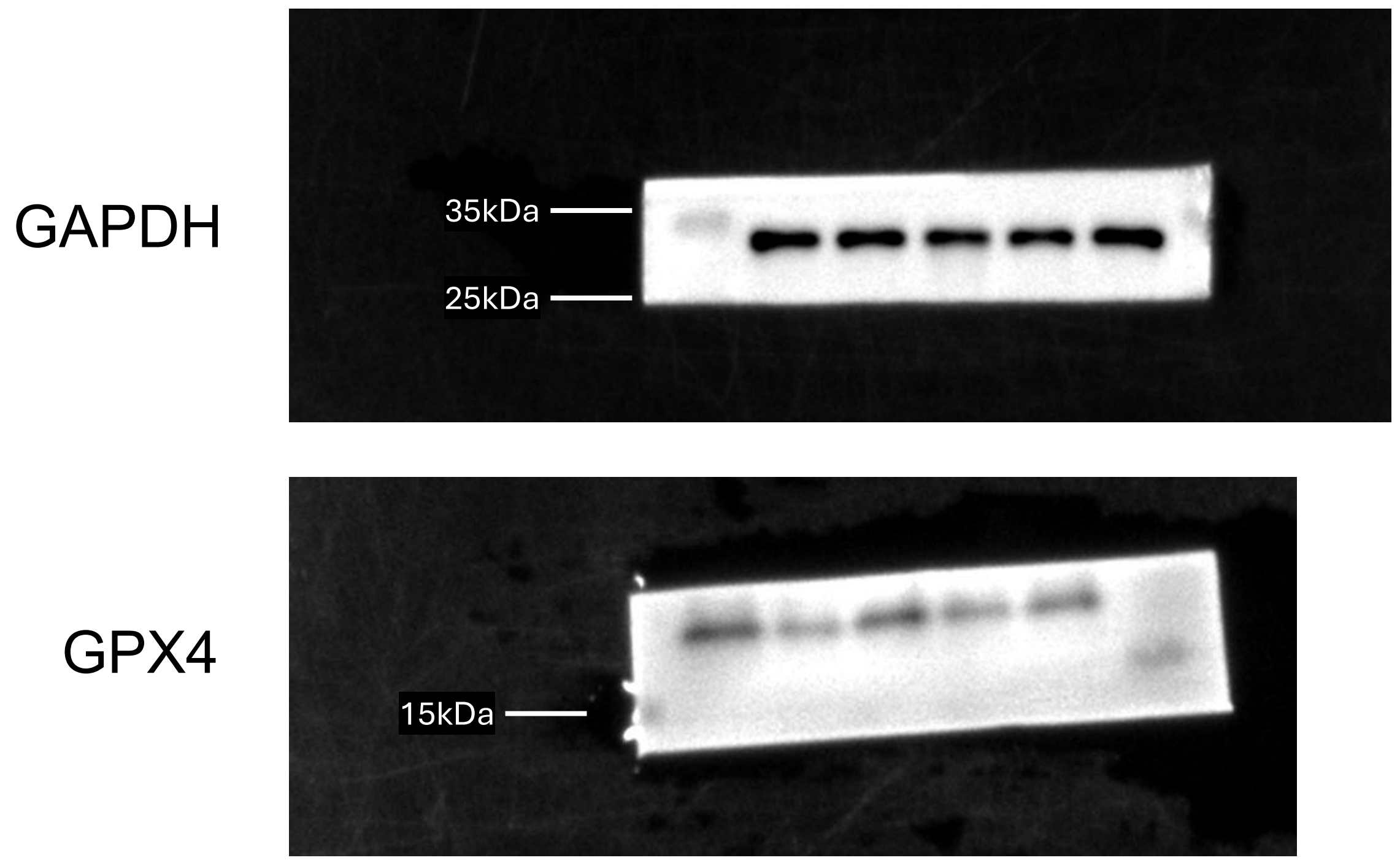
GAPDH
35kDa
25kDa
GPX4
15kDa

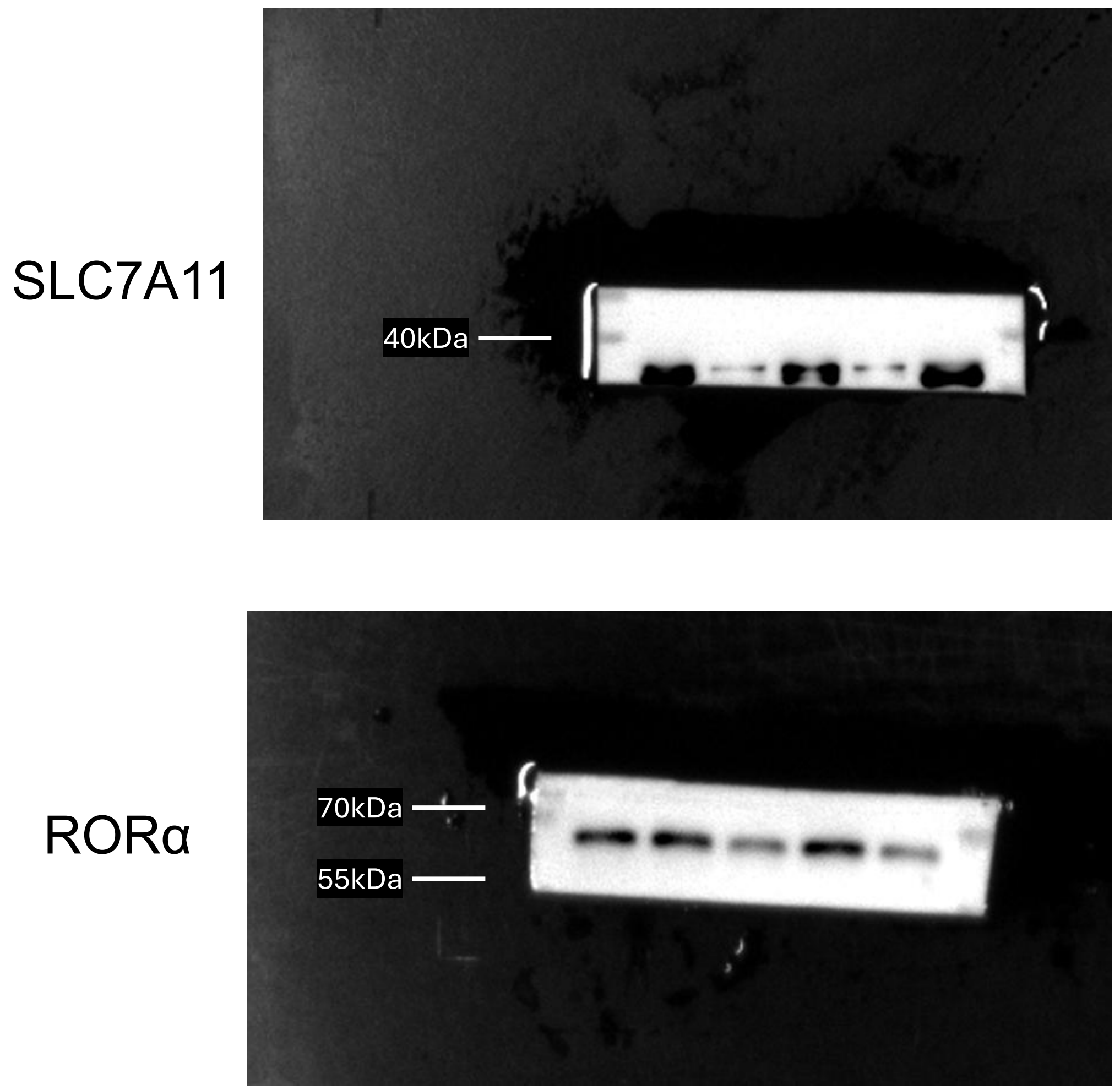
SLC7A11
40kDa
RORα
70kDa
55kDa

Fig5 A

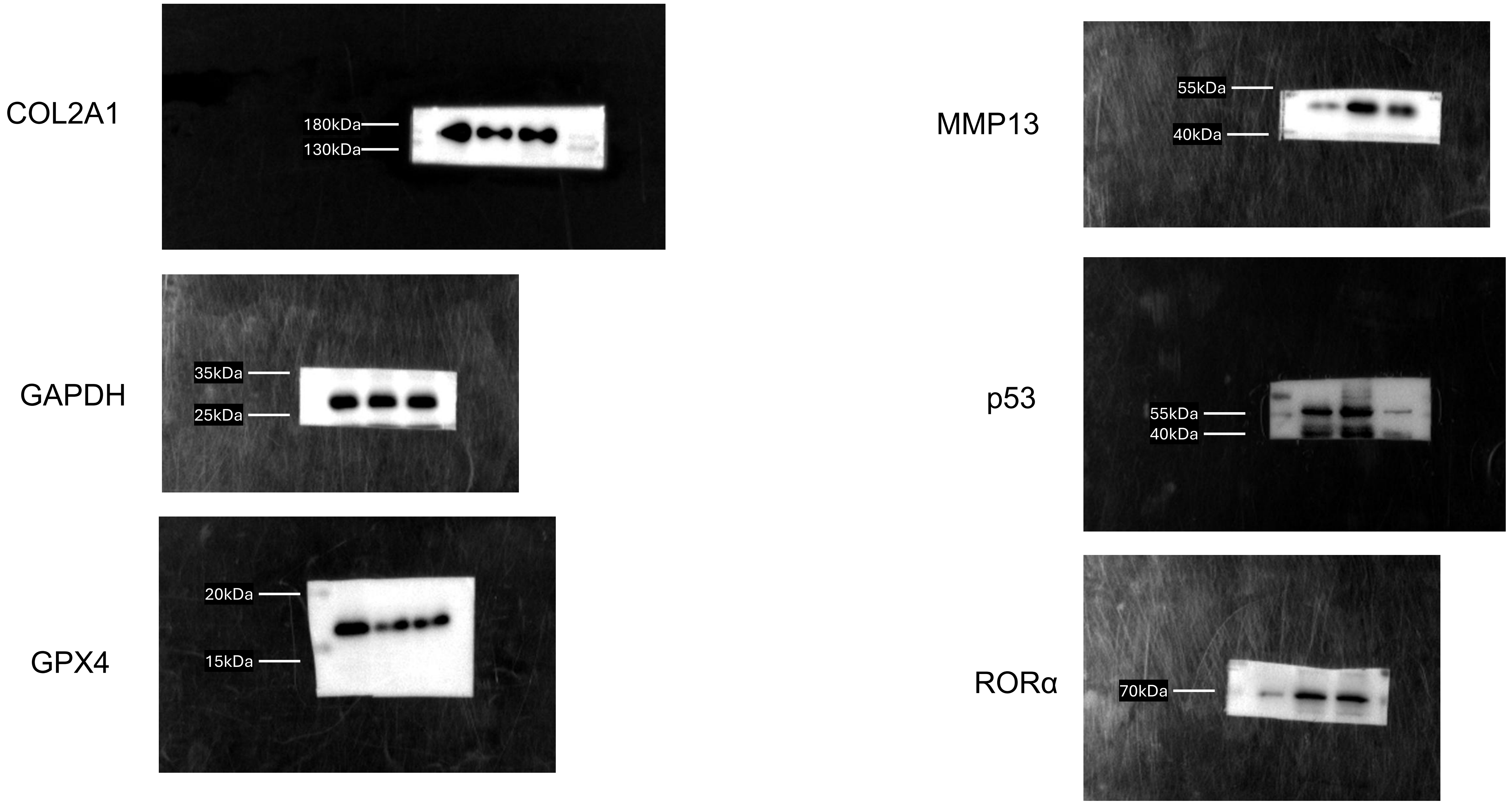

COL2A1
180kDa
130kDa
GAPDH
35kDa
25kDa
GPX4
20kDa
15kDa
MMP13
55kDa
40kDa
p53
55kDa
40kDa
RORα
70kDa

Fig6 A

Input: RORα

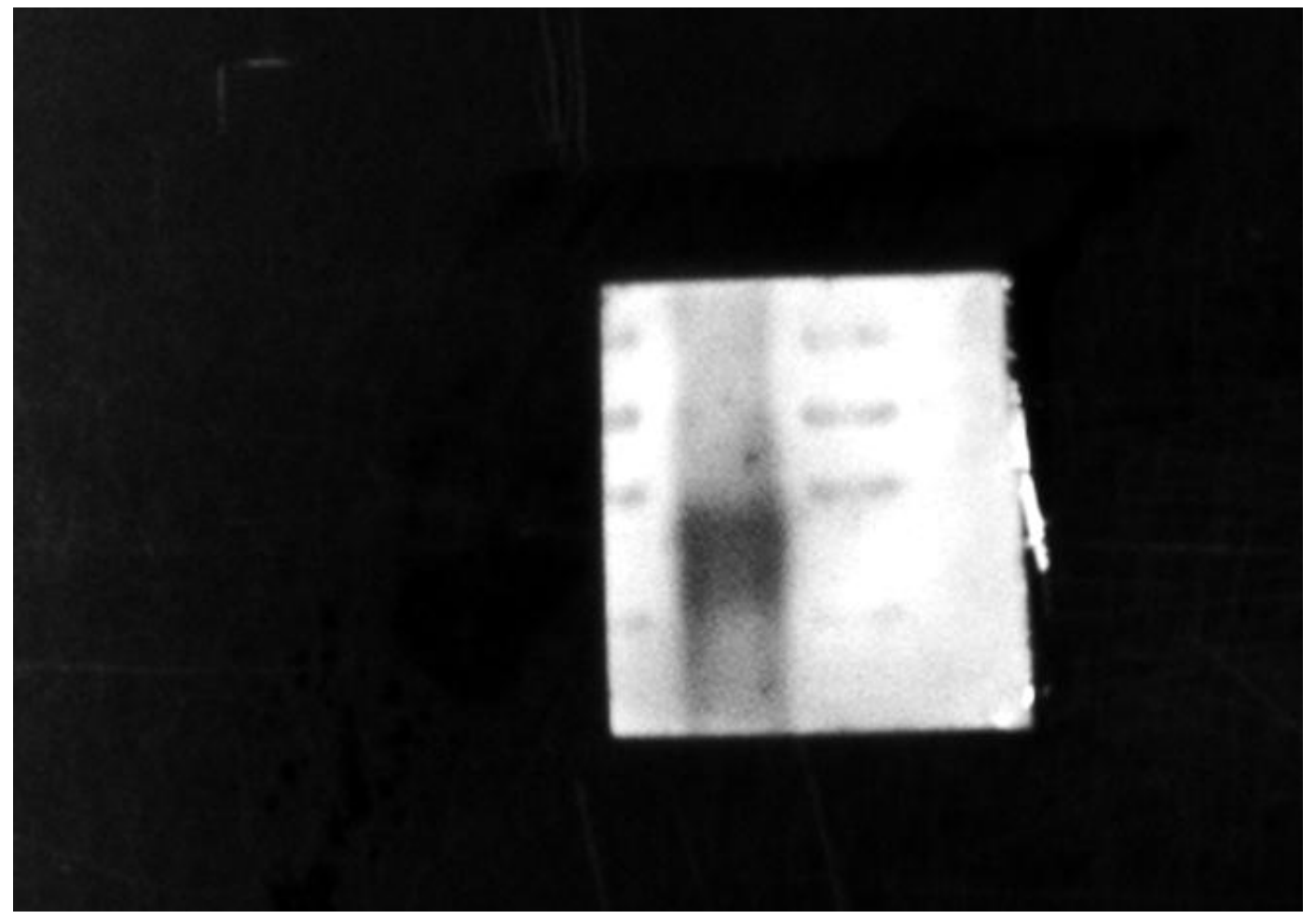

Input: p53

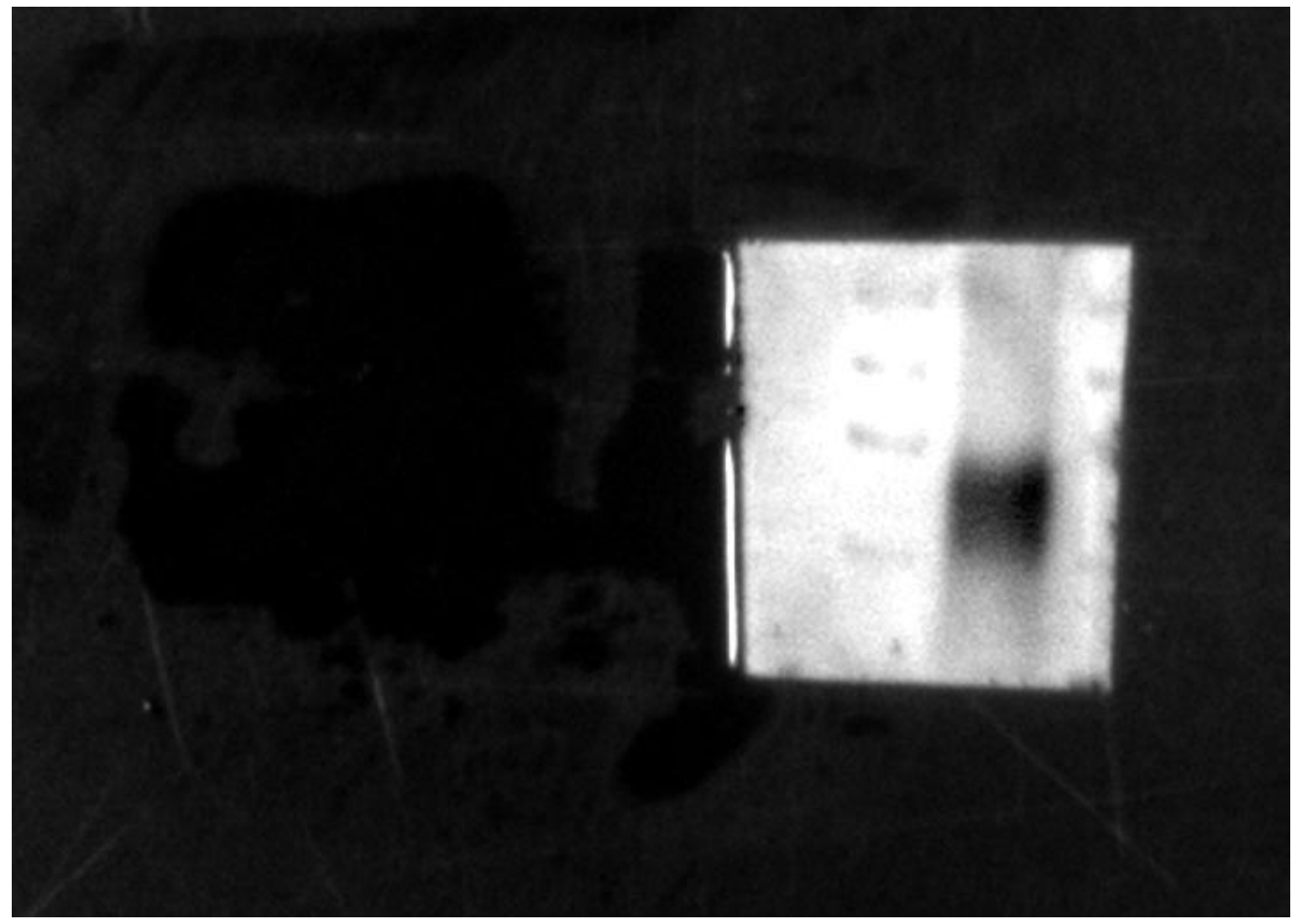

IB: RORα

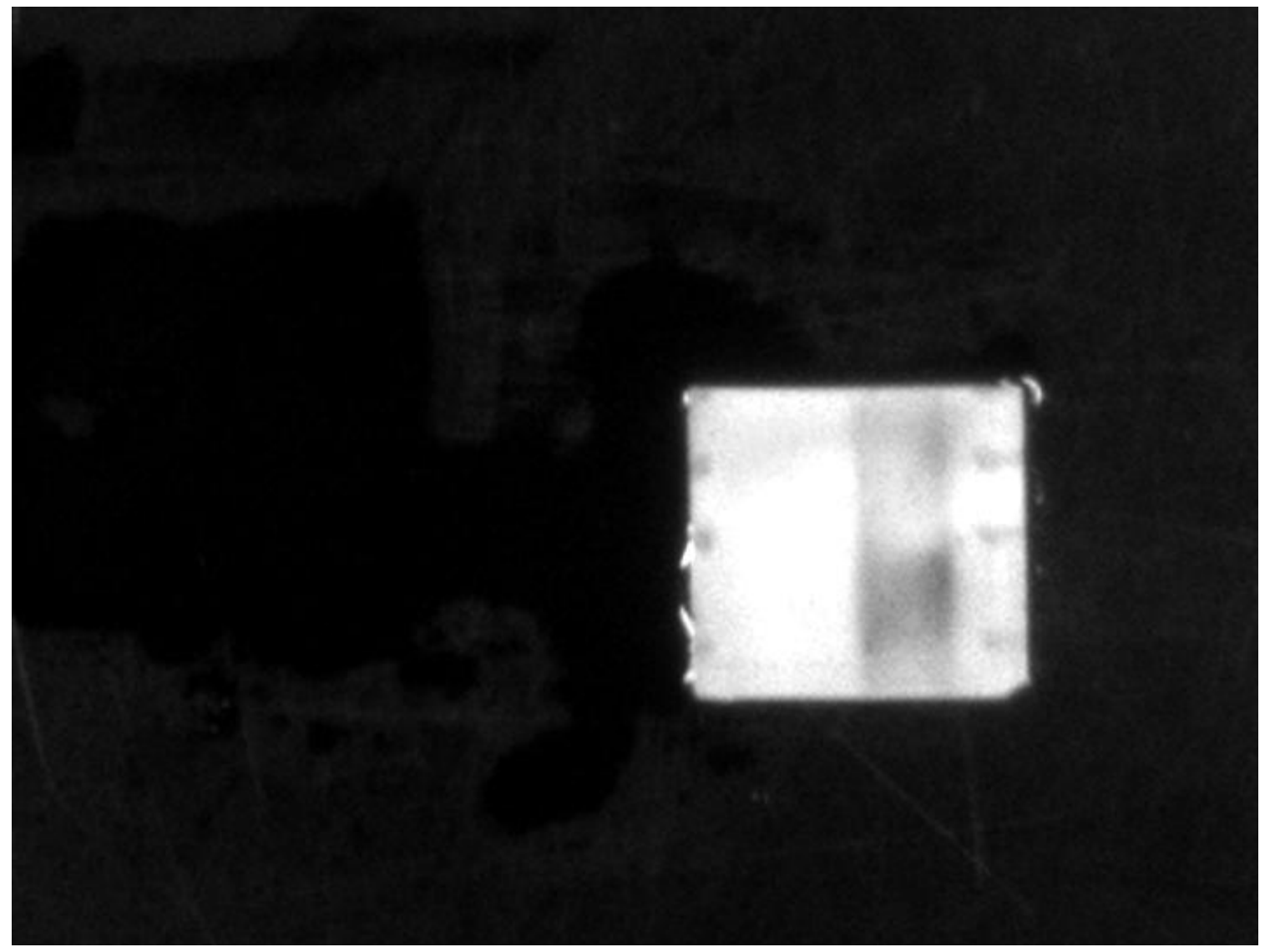

IB: p53

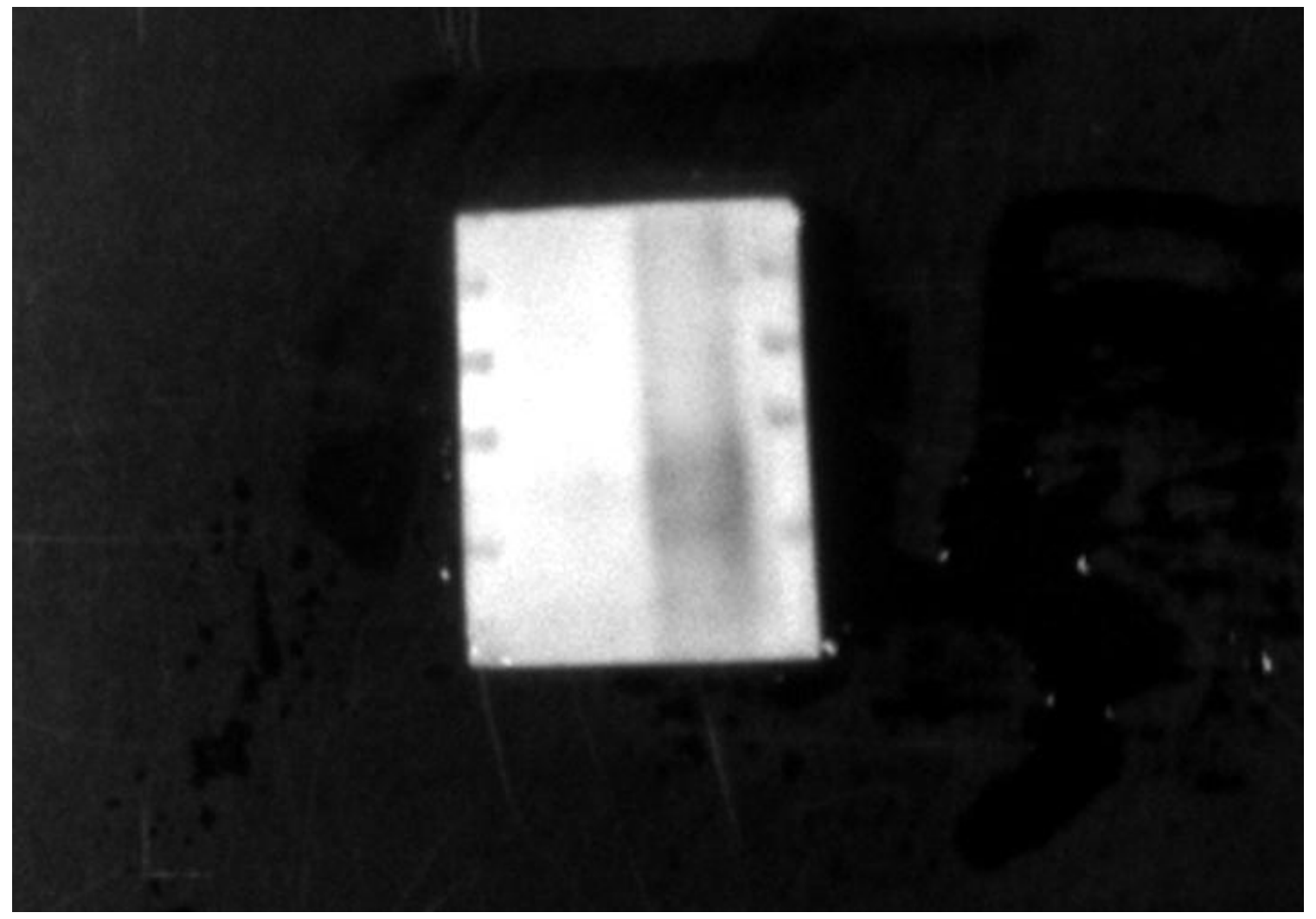

Fig6 B

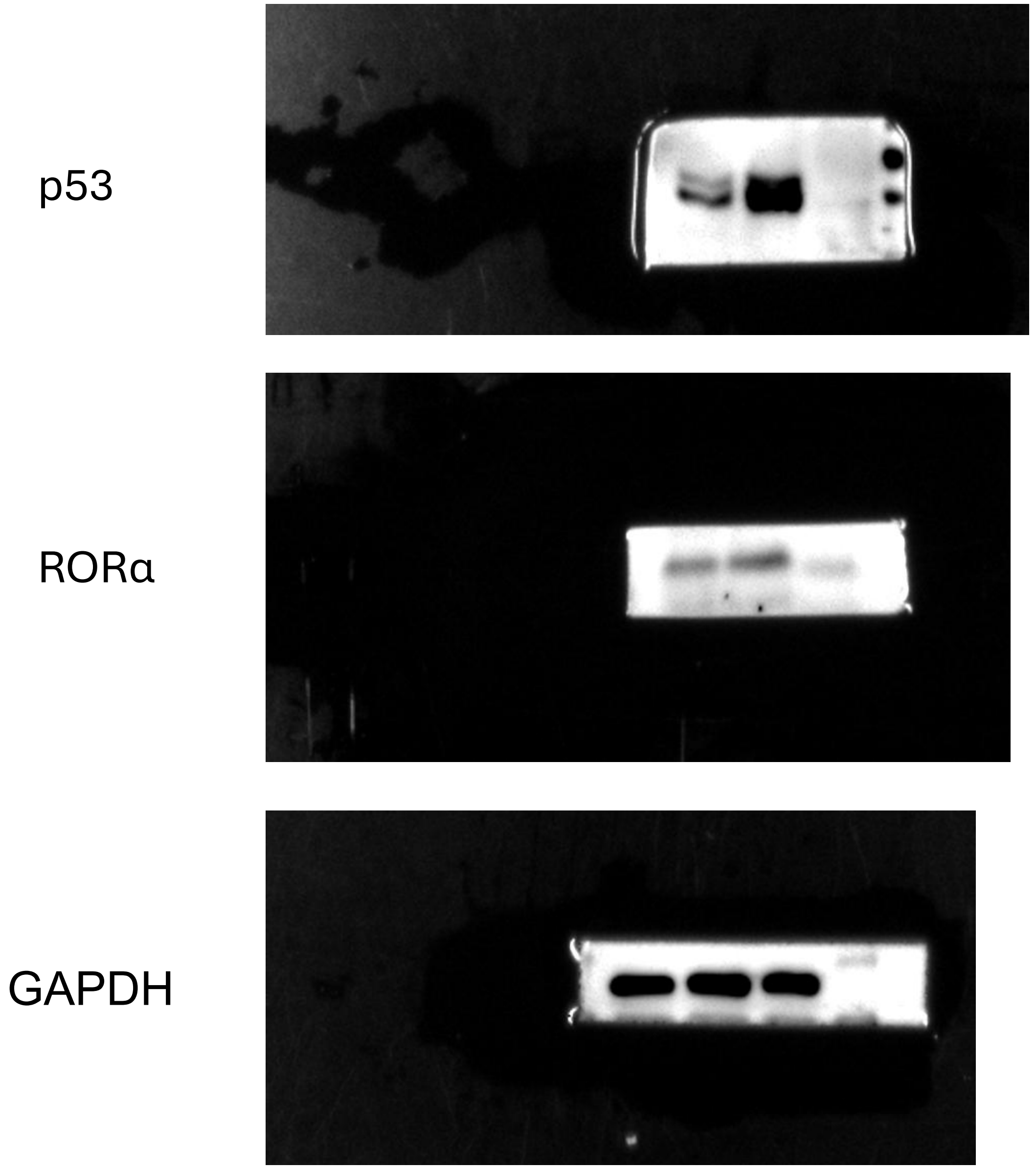

Fig6 C

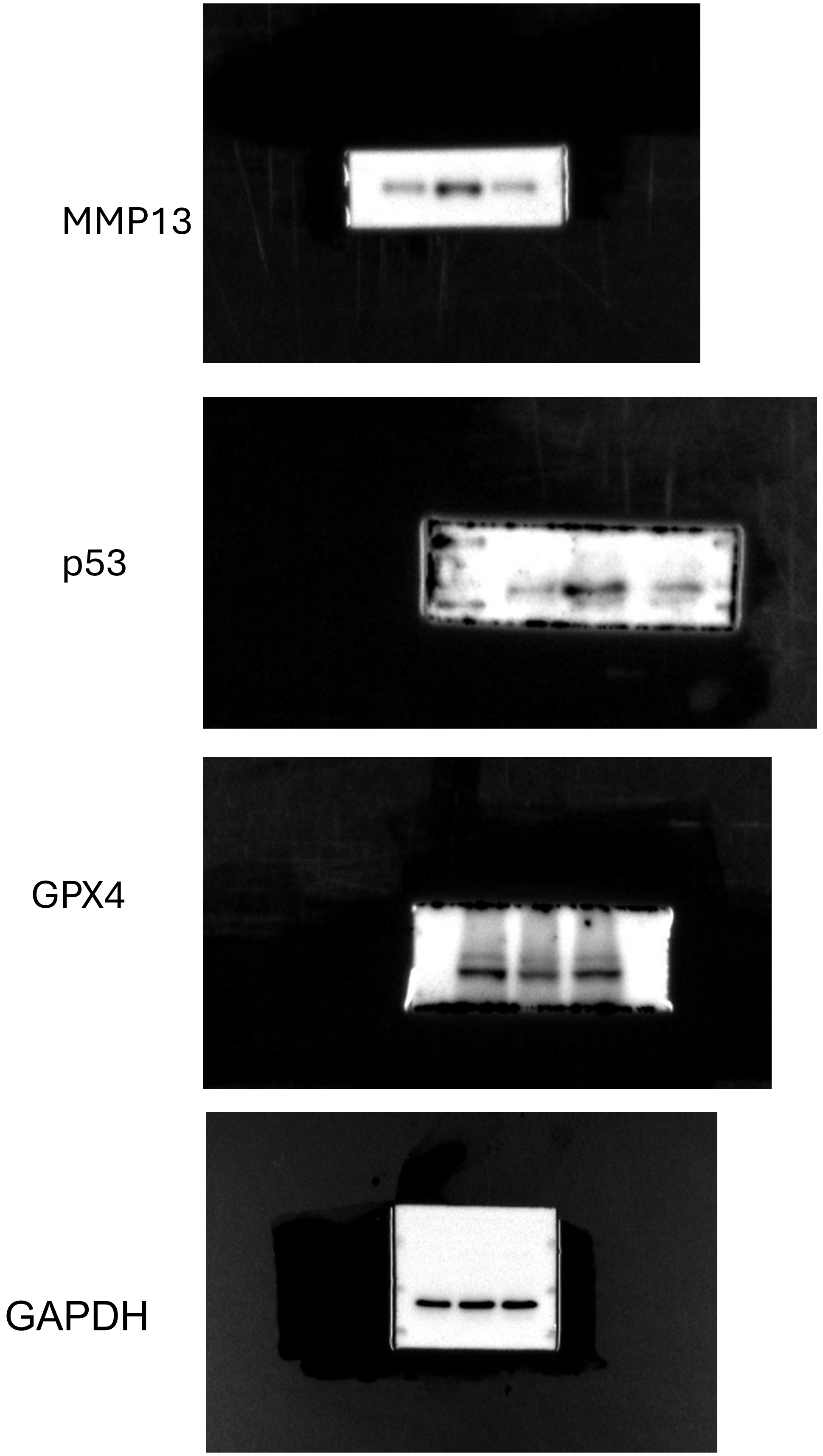

Fig6 D

HAUSP

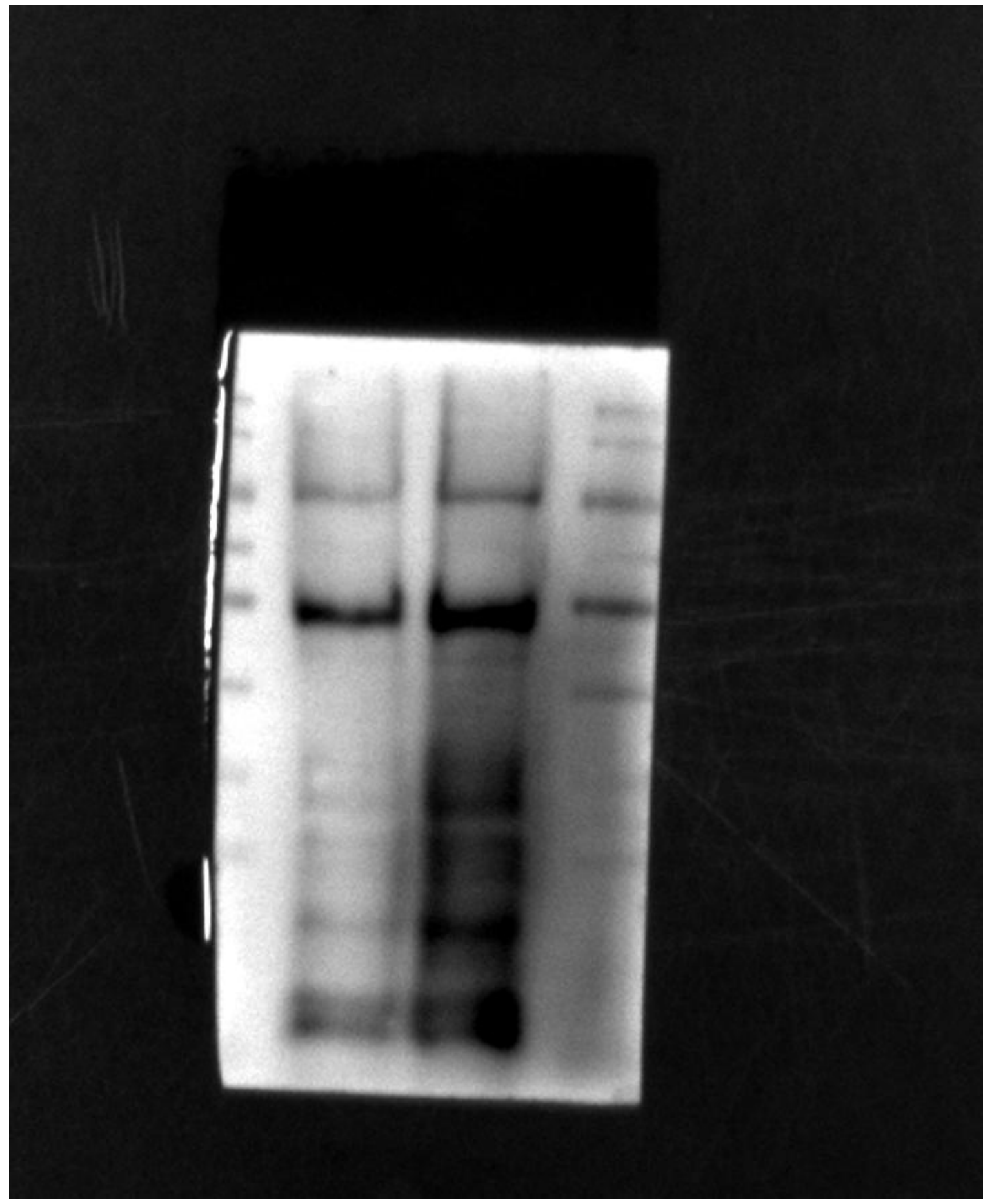

Input: p53

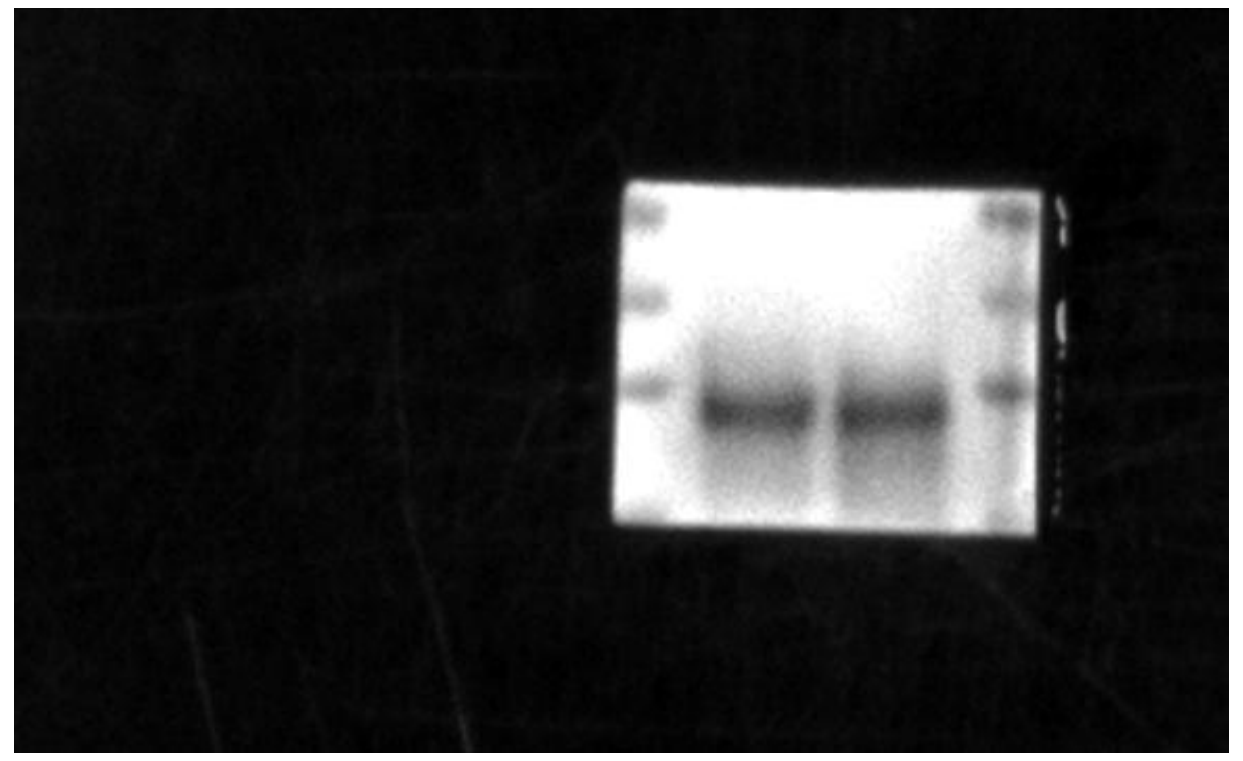

p53

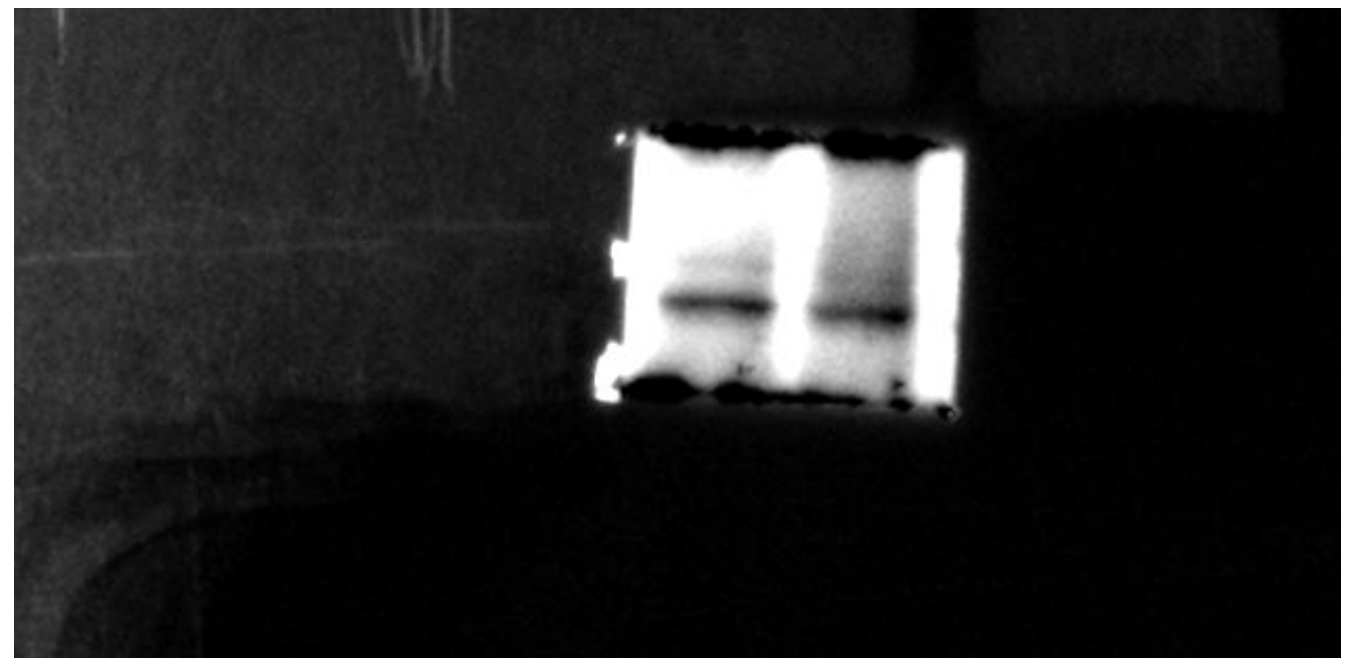

Input:
RORα

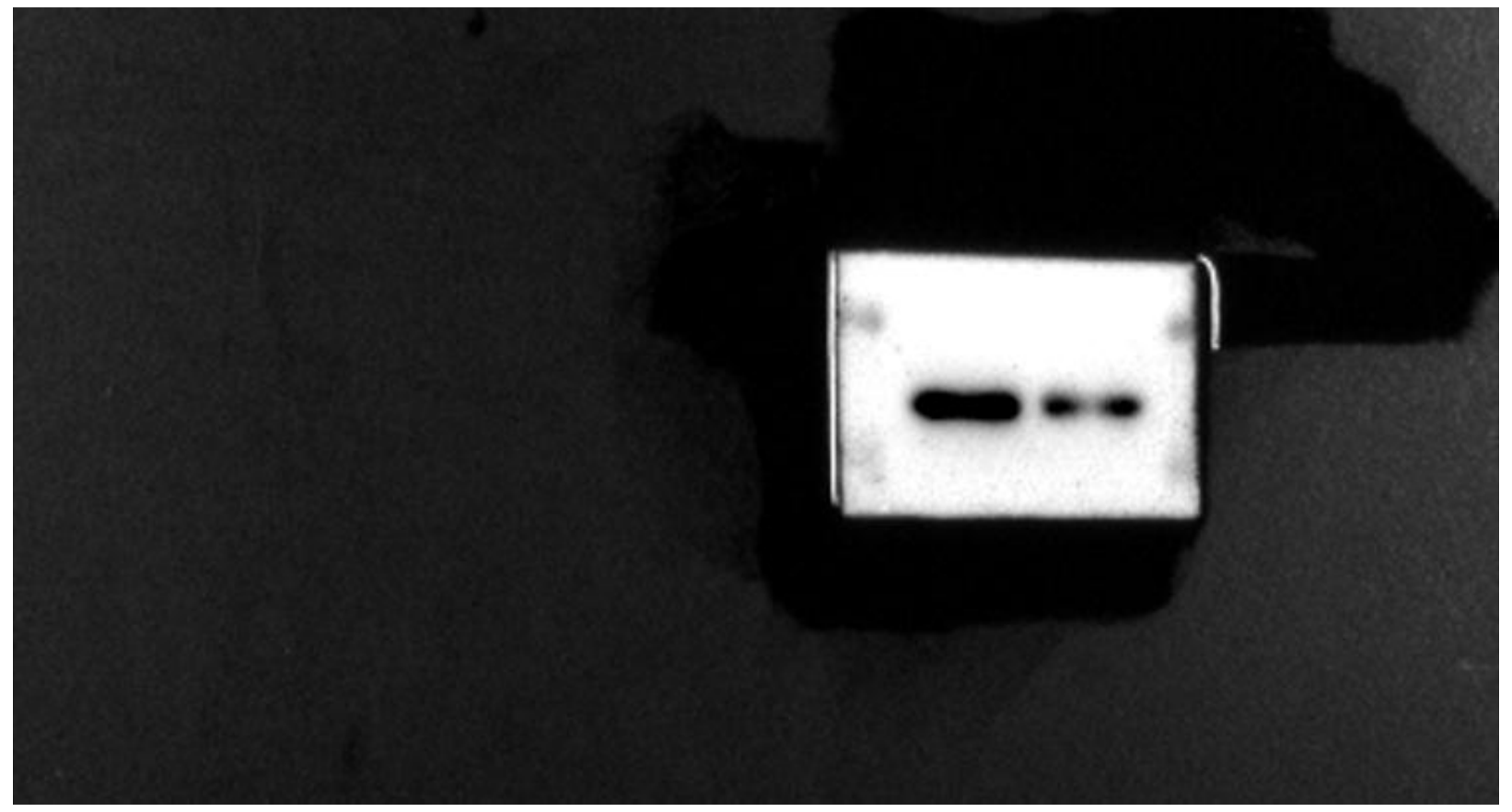

Fig6 E

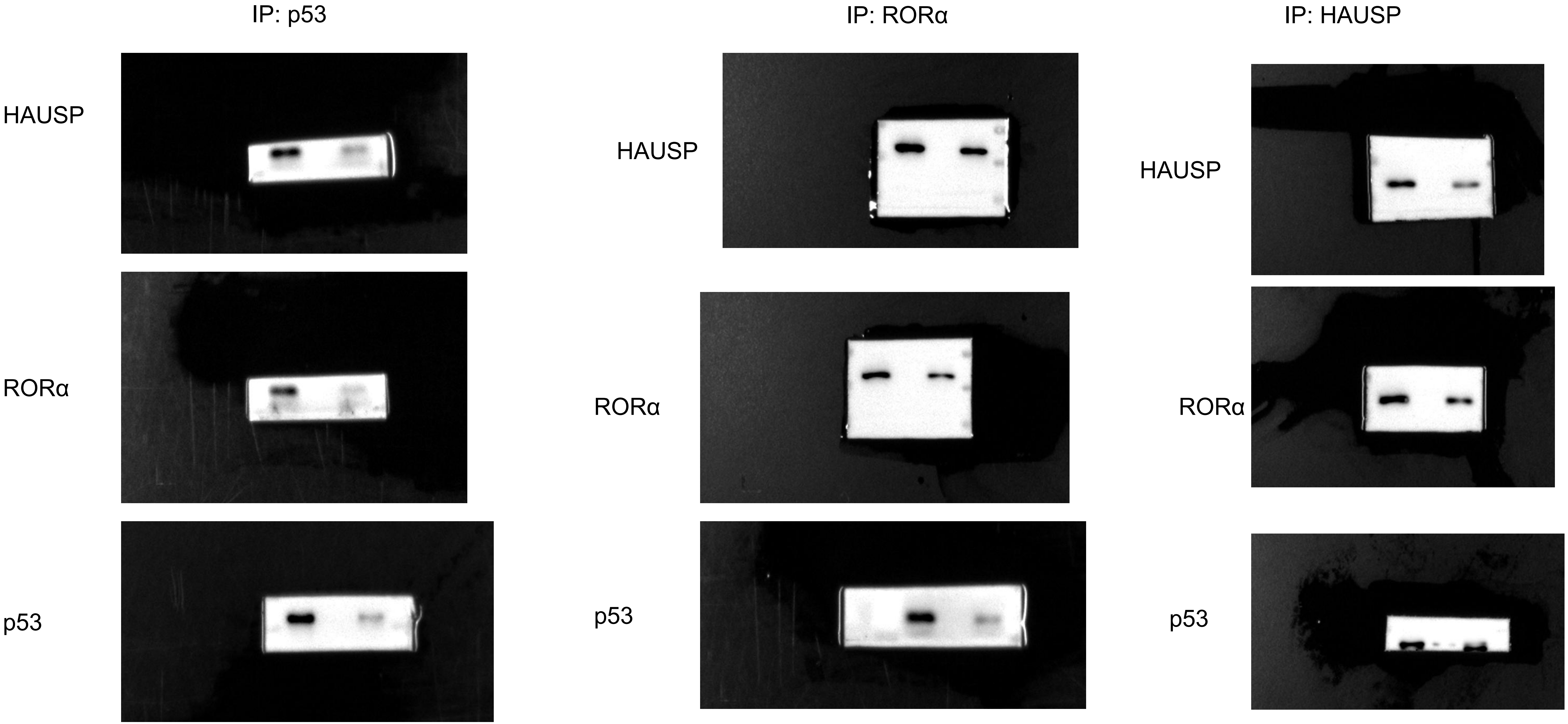
IP: p53
IP: RORα
IP: HAUSP
HAUSP
RORα
p53
HAUSP
RORα
p53
HAUSP
RORα
p53

Fig6 F

Input
p53

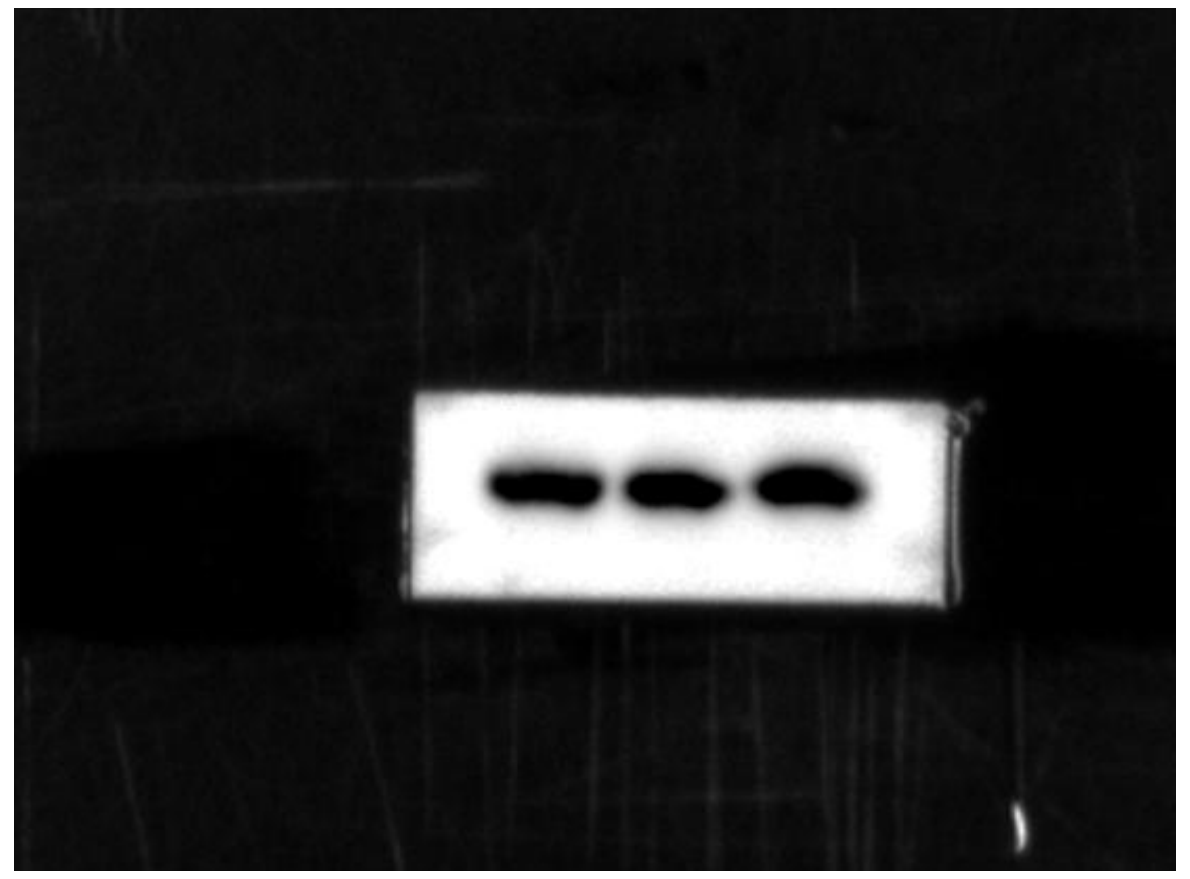

Input
RORα

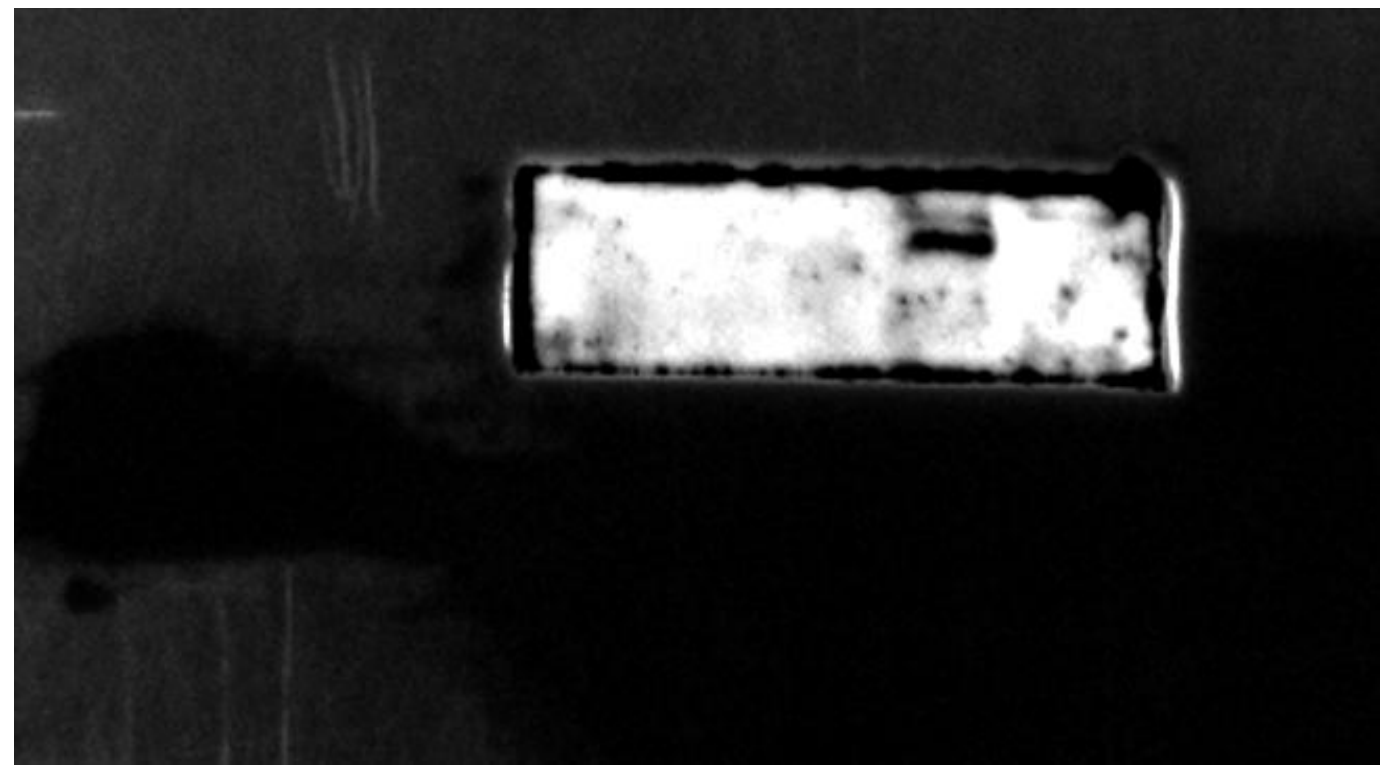

Input
HAUSP

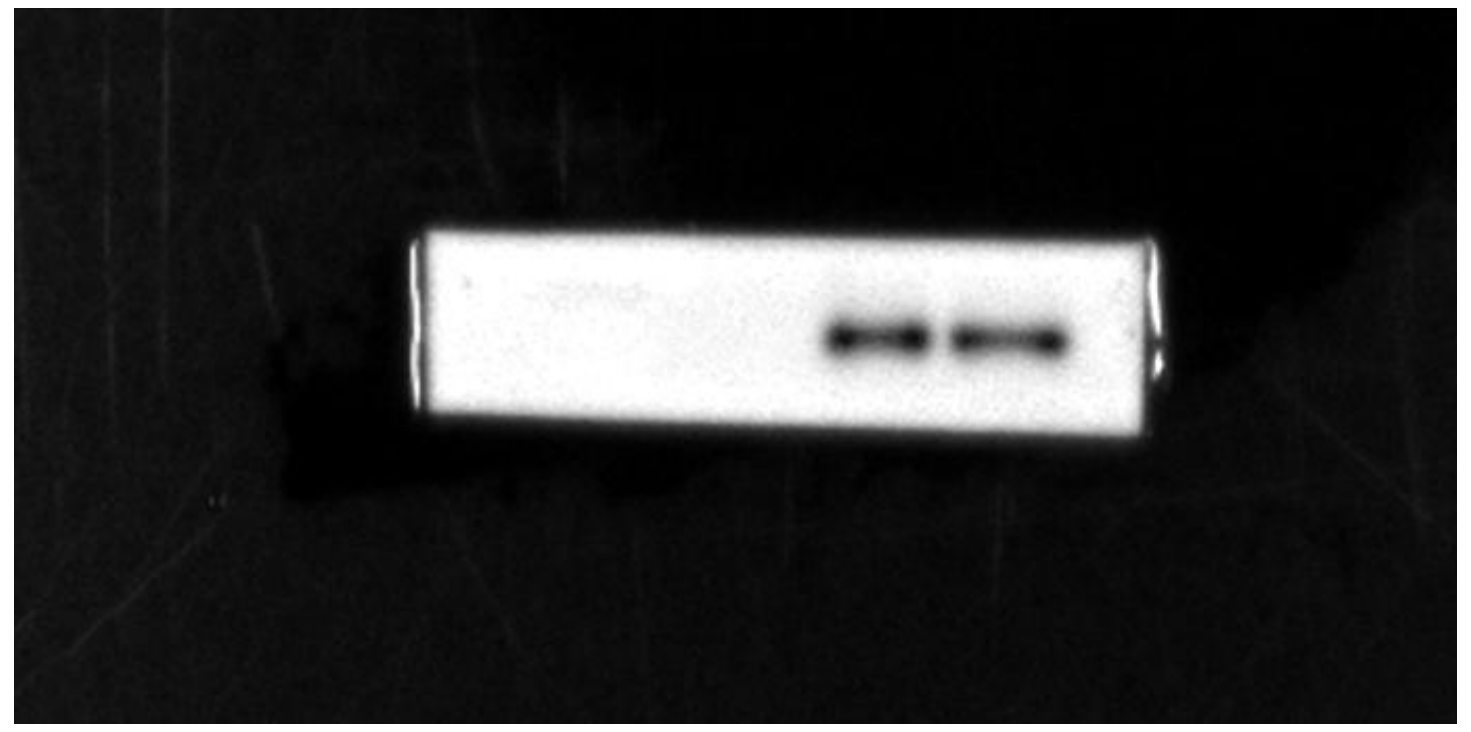

p53

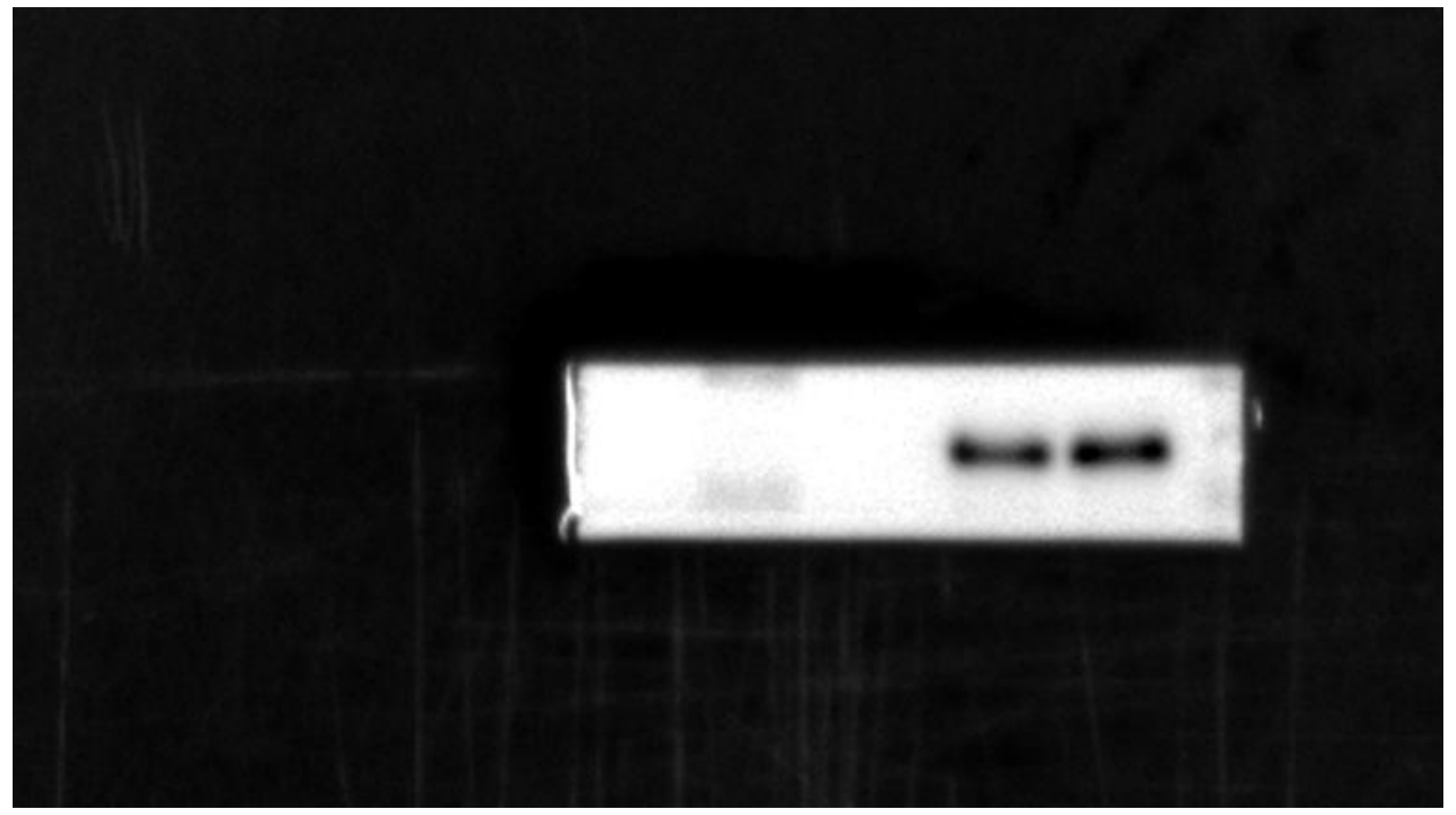

HAUSP

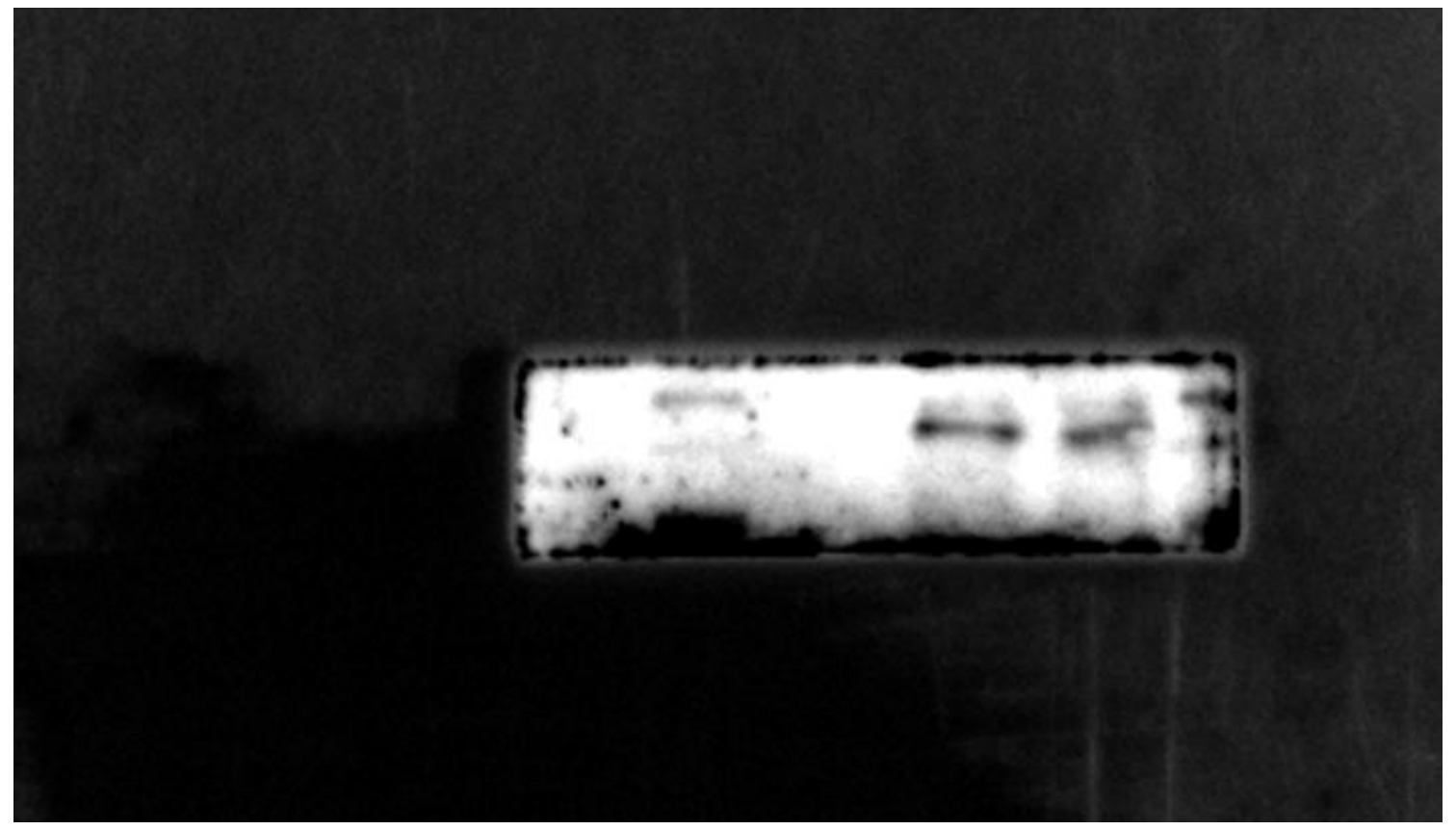

Fig6 G

Input
RORα

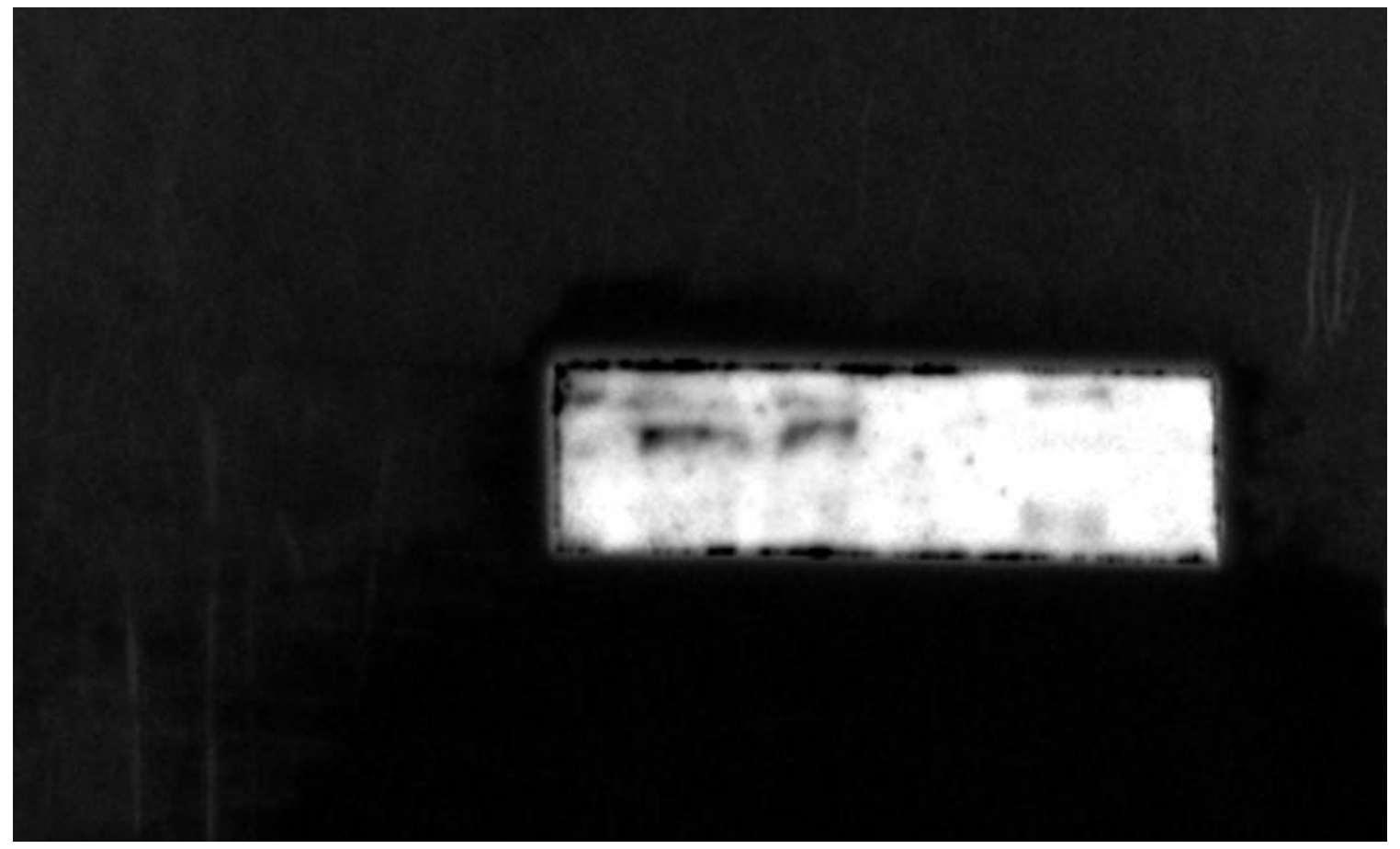

Input
p53

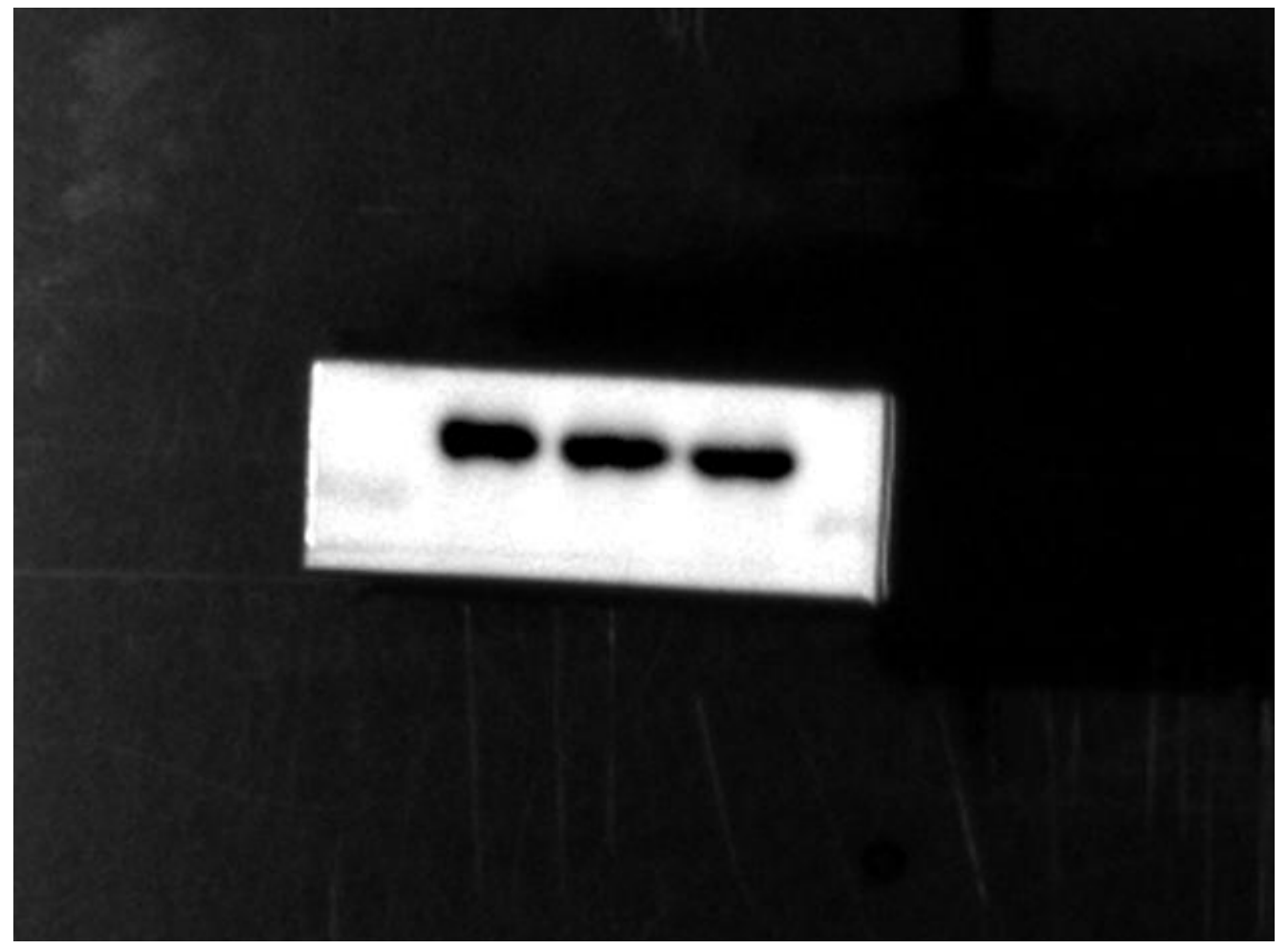

p53

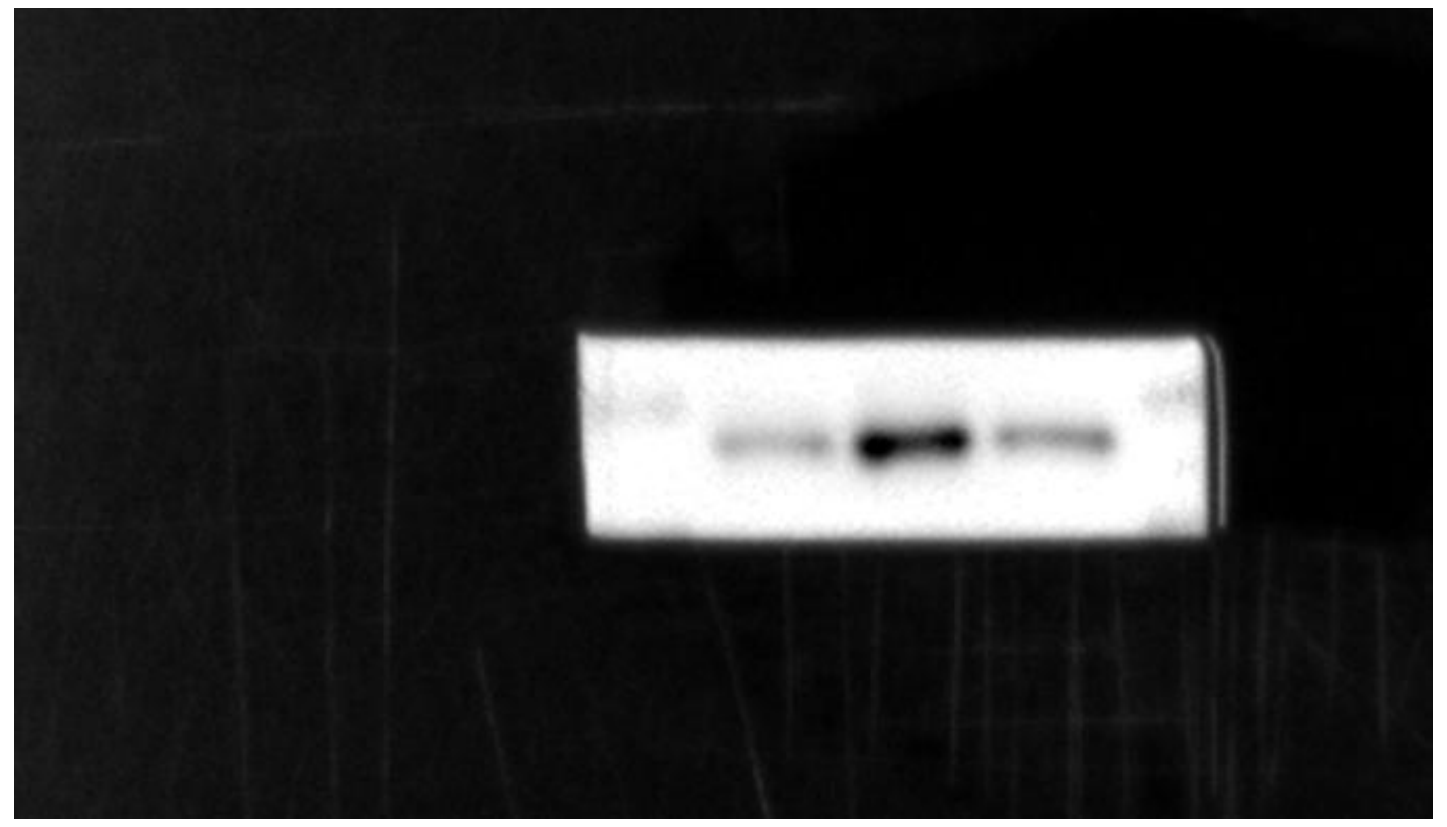

Input
HAUSP

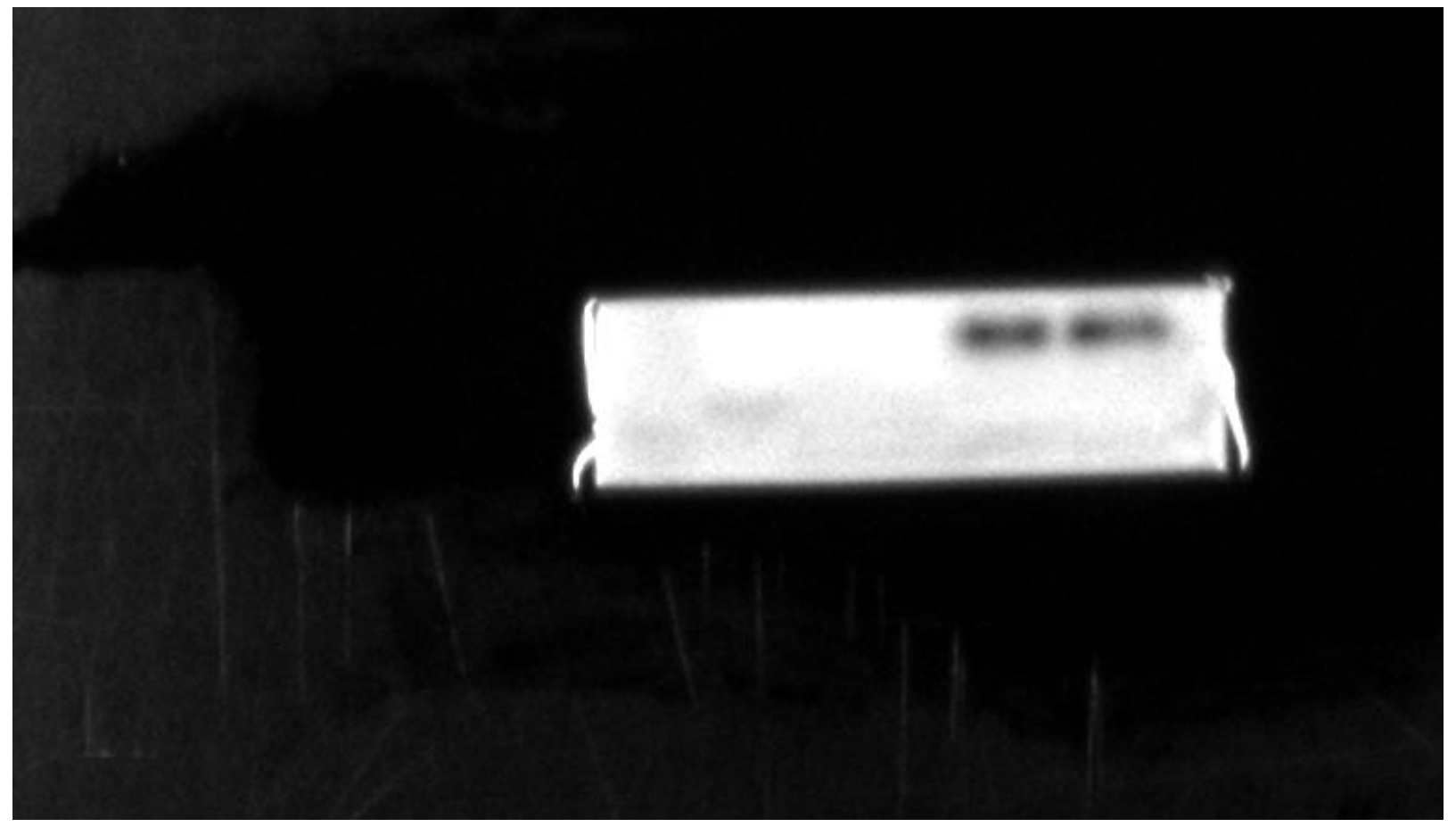

HAUSP

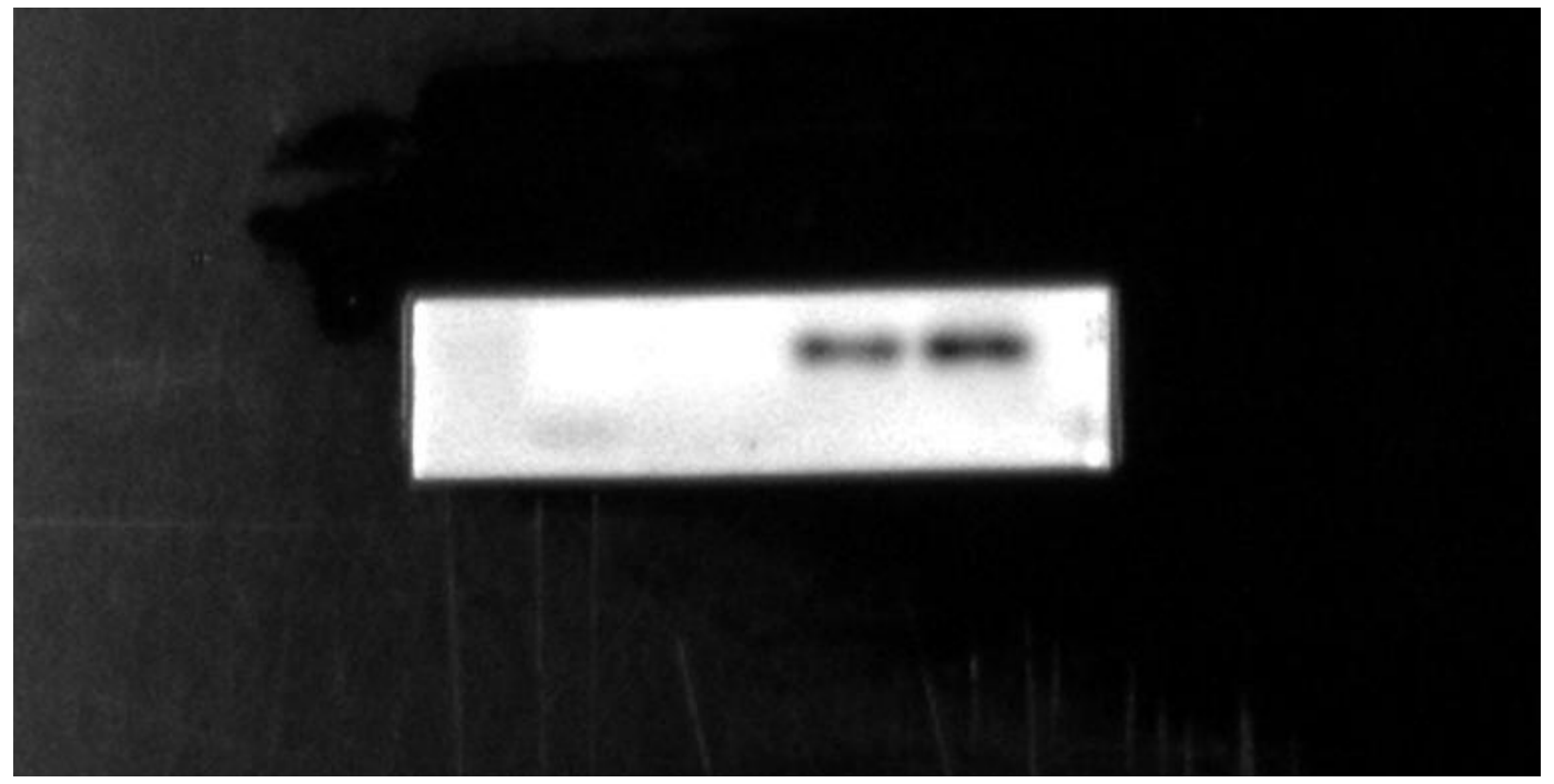

Supplement: Supplementary file 1 [file mmc1.pdf]
